# Sex-specific impact of diabetes on all-cause mortality among adults with acute myocardial infarction: An updated systematic review and meta-analysis, 1988-2021

**DOI:** 10.3389/fendo.2022.918095

**Published:** 2022-08-17

**Authors:** Qinglan Ding, Marjorie Funk, Erica S. Spatz, Haiqun Lin, Janene Batten, Emily Wu, Robin Whittemore

**Affiliations:** ^1^ College of Health and Human Sciences, Purdue University, West Lafayette, IN, United States; ^2^ School of Nursing, Yale University, West Haven, CT, United States; ^3^ Center for Outcomes Research and Evaluation, Yale-New Haven Hospital, New Haven, CT, United States; ^4^ Section of Cardiovascular Medicine, Department of Internal Medicine, Yale School of Medicine, New Haven, CT, United States; ^5^ Rutgers University School of Nursing, Newark, NJ, United States; ^6^ Harvey Cushing/John Hay Whitney Medical Library, Yale University, New Haven, CT, United States; ^7^ Krannert School of Management, Purdue University, West Lafayette, IN, United States

**Keywords:** diabetes mellitus, sex-specific, acute myocardial infarct (AMI), meta-analysis, systemic review, all-cause mortality

## Abstract

**Background:**

The prevalence of diabetes and its impact on mortality after acute myocardial infarction (AMI) are well-established. Sex-specific analyses of the impact of diabetes on all-cause mortality after AMI have not been updated and comprehensively investigated.

**Objective:**

To conduct a systematic review and meta-analysis that examined sex-specific short-term, mid-term and long-term all-cause mortality associated with diabetes among AMI survivors (diabetes versus non-diabetes patients in men and women separately), using up-to-date data.

**Methods:**

We systematically searched Embase and MEDLINE for studies that were published from inception to November 14, 2021. Studies were included if (1) they studied post-AMI all-cause-mortality in patients with and without diabetes, (2) sex-specific all-cause mortality at short-term (in-hospital or within 90 days after discharge), mid-term (>90 days and within 5 years), and/or long-term (>5 years) were reported. From eligible studies, we used random effects meta-analyses models to estimate pooled unadjusted and adjusted sex-specific risk ratio (RR) of all-cause mortality at short-, mid-, and long-term follow-up for adults with diabetes compared with those without diabetes.

**Results:**

Of the 3647 unique studies identified, 20 studies met inclusion criteria. In the unadjusted analysis (Total N=673,985; women=34.2%; diabetes patients=19.6%), patients with diabetes were at a higher risk for all-cause mortality at short-term (men: RR, 2.06; women: RR, 1.83); and mid-term follow-up (men: RR, 1.69; women: RR, 1.52) compared with those without diabetes in both men and women. However, when adjusted RRs were used (Total N=7,144,921; women=40.0%; diabetes patients=28.4%), the associations between diabetes and all-cause mortality in both men and women were attenuated, but still significantly elevated for short-term (men: RR, 1.16; 95% CI, 1.12-1.20; women: RR, 1.29; 95% CI, 1.15-1.46), mid-term (men: RR, 1.39; 95% CI, 1.31-1.46; women: RR, 1.38; 95% CI, 1.20-1.58), and long-term mortality (men: RR, 1.58; 95% CI, 1.22-2.05; women: RR, 1.76; 95% CI, 1.25-2.47). In men, all-cause mortality risk associated with diabetes tended to increase with the duration of follow-up (p<0.0001).

**Conclusions:**

Diabetes has substantial and sustained effects on post-AMI all-cause mortality at short-term, mid-term and long-term follow-up, regardless of sex. Tailoring AMI treatment based on patients’ diabetes status, duration of follow-up and sex may help narrow the gap in all-cause mortality between patients with diabetes and those without diabetes.

## Introduction

Diabetes mellitus is a well-established risk factor for cardiovascular disease (CVD) and is associated with higher rates of acute myocardial infarction (AMI), as well as an increased risk of mortality after AMI than in adults without diabetes (1–3). The excess risk associated with diabetes is explained by a complex combination of various traditional (hypertension, hyperlipidemia, obesity, etc.) and non-traditional cardiometabolic risk factors (insulin resistance, glucose variability, genetics, etc.) that have important roles in the pathogenesis of atherosclerosis and cardiovascular complications (2, 4). Accumulating evidence also suggests significant sex differences concerning the impact of diabetes on post-AMI mortality (5–7). The excess risks of adverse outcomes after AMI attributable to diabetes appear to be more potent in women than in men. Women with diabetes have a three-fold greater risk for fatal coronary heart disease compared with women without diabetes, and have a higher adjusted hazard ratio of fatal coronary heart disease compared with men with diabetes (8). But although some studies largely support this observation, some do not (9, 10). A pooled analysis combining all available evidence reporting the sex-specific impact of diabetes on short-term, mid-term and long-term mortality among patients with AMI is lacking in the literature (11). It is not quite clear whether the observed sex-specific impact of diabetes on post-AMI mortality is due to differences in duration of follow-up, and/or differences in study populations and methods (12), or if this is a confounded observation due to baseline differences in characteristics between patients with and without diabetes, such as differences in traditional cardiometabolic risk factors (2, 13).

This review presents an update of the evidence to the present time as the last two meta-analyses on a similar topic were performed in early 2000 (13, 14). Both previous meta-analyses assessed the impact of diabetes on coronary heart disease (CHD) mortality (including CHD mortality after AMI) in men and women (13, 14). The meta-analysis published in 2000 included 10 prospective cohort studies that provided data to pool the adjusted sex-specific relative risk of CHD mortality associated with diabetes. The risk of fatal CHD increased for both diabetic women and diabetic men, compared to their sex-specific counterparts without diabetes (RRs 2.58 for women, 1.85 for men) (14). Based on the calculated adjusted sex-specific RR of CHD mortality (diabetes-to-non-diabetes), the study concluded a significantly greater risk of CHD death attributable to diabetes for women than men. The meta-analysis published in 2002 estimated summary odds ratios associated with diabetes for all-cause mortality besides CHD mortality (13). This meta-analysis included 8 studies and found little difference between summary odds ratios for all-cause mortality due to diabetes between men and women (Summary Odds in men with diabetes vs. men without diabetes: 2.1, Odds in women with diabetes vs. women without diabetes: 1.9). Additionally, this more recent meta-analysis noticed heterogeneity among included studies could not be eliminated by subgroup analysis (by race or study design). Despite a wide range of follow-up duration among the included studies in the two prior meta-analyses (meta-analysis in 2000: follow-up ranged from 9-20 years, meta-analysis in 2002: ranged from 5-32 years), neither pooled their estimates attributable to diabetes separately for short-, mid- and long-term follow-up. Further, recent advances in the treatment of AMI and new antidiabetic medications have dramatically reduced mortality and improved outcomes of patients with diabetes (11, 15, 16). It remains unclear whether the reported greater impact of diabetes on post-AMI all-cause mortality for women has changed over recent years. Therefore, it is necessary to include more up-to-date data and reassess the sex-specific impact of diabetes on mortality after AMI.

The goal of the study was to quantify risk for post-AMI all-cause mortality associated with diabetes in men and women. Specifically, we report sex-specific summary estimates of the risk ratios (RRs) of short-term (defined as in-hospital or within 90 days after discharge), mid-term (defined as >90 days and within 5 years), and long-term (defined as >5 years) all-cause mortality for adults with diabetes compared with those without diabetes (RRs for men with diabetes versus men without diabetes and RRs for women with diabetes-to-women without diabetes). We conducted a systematic review and meta-analysis on relevant randomized clinical trials and observational studies and included more recent publications. Additionally, we assessed whether the trend of short-term, mid-term, and long-term estimates of RRs of post-AMI all-cause mortality associated diabetes over time was different between men and women.

## Methods

### Search strategy, data sources, and selection criteria

This meta-analysis was conducted in accordance with the Preferred Reporting Items for Systematic Reviews and Meta-analysis (PRISMA) guidelines (17). Using *a priori* established inclusion and exclusion criteria (see below), two authors (QD and JB) independently conducted a comprehensive literature search in two databases (including OVID EMBASE and OVID MEDLINE) initially on February 21, 2017. The search strategy was first developed by our medical librarian (JB) in 2017 and later peer-reviewed by another librarian (JY) in 2021. Using the peer-reviewed search strategy, JB re-ran the search in the two databases periodically to help identify more recent studies (the most recent search was performed on November 14, 2021).

The search was done using controlled vocabulary terms and free-text words to capture the concept of *outcome* or *survival* or *mortality* or *prognosis* in *men* and *women with diabetes* and *myocardial infarction* or *heart attack* or *acute coronary syndrome*. The search strategies were adjusted for the syntax appropriate for each database/platform. The search was limited to articles written in English. We identified additional studies by examining reference lists of selected articles. Search terms and detail search strategy are detailed in the Supplementary Data.

**Figure 1:**
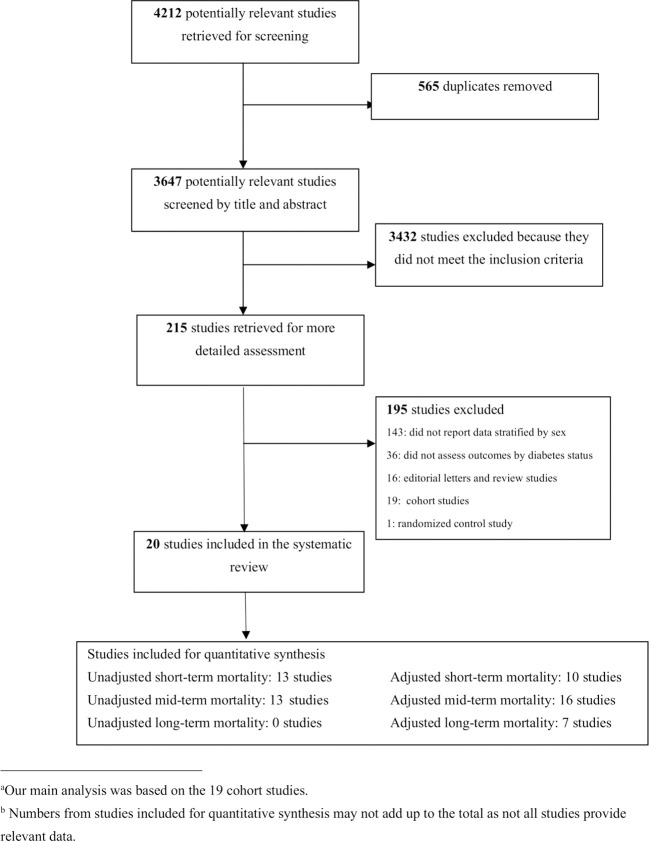
Flow diagram of study for review based on inclusion and exclusion criteria^ab^.

All published studies that evaluated the occurrence of short-, mid- and long-term all-cause mortality in adults with and without diabetes after AMI were identified. Studies were included if they met the following criteria: (1) the study population included patients with AMI without diabetes, in addition to patients with diabetes (≥18 years); (2) sex-specific short-, mid- and/or long-term all-cause mortality after AMI was reported for patients with and without diabetes. If a study did not provide sex-specific RRs, odds ratio (ORs), or hazard ratio of post-AMI all-cause mortality, but provided sufficient information (e.g., number of men or women with and without diabetes and group-specific mortality) for researchers to calculate unadjusted RR by sex, this study was also included. We excluded review articles, editorials, case-reports, duplicate studies, unpublished manuscripts, and electronic articles in non-journal formats. **Figure 1** illustrates the study selection process.

### Data extraction

Two investigators (QD and EW) independently reviewed the studies and extracted data using a standardized data extraction form. Disagreement was resolved by consensus. Extracted studies included randomized clinical trials and observational studies. We collected data on study lead authors, publication year, country, study design (observational or randomized controlled trial), follow-up period, sample size, age by sex and diabetes status, number of patients by sex and by diabetes status, number of patients who died after AMI by sex and diabetes status. Whenever available, we also extracted data regarding potential confounding variables that were adjusted for by the included studies. The following data was also collected from each study for sex-specific diabetic and non-diabetic groups: adjustment types, unadjusted and adjusted post-AMI all-cause mortality RRs and 95% confidence intervals (CIs). We used RRs instead of ORs because when the outcome is not rare, ORs tend to overestimate the strength of association (18). When ORs for post-AMI all-cause mortality were reported instead of RRs, the unadjusted RRs were calculated from the study sample size, number of men or women with and without diabetes, the sex-specific crude post-AMI all-cause mortality rates by diabetes status using a R package (orsk) (19).

### Quality appraisal and heterogeneity

National Heart, Lung, and Blood Institute’s Quality Assessment Tool for Observational Cohort and Cross-Sectional Studies (20) was used to evaluate the quality of the included 19 observational studies (21). The Revised Cochrane risk-of-bias tool for randomized trials (RoB2) (22) was used for quality assessment of the single randomized controlled trial.

Heterogeneity across studies was assessed using the Cochran Q statistic (χ^2^) and *I* ^2^ statistics. We classified between-study heterogeneity into the following category: low heterogeneity (*I* ^2^ <25%), moderate (*I* ^2 ^= 25% to 50%), substantial (*I* ^2^ >50%) (23). In case of high heterogeneity, we performed multiple subgroup analyses, with the removal of one or more studies, to identify the source of heterogeneity. The risk of publication bias was evaluated by funnel plots (**Supplementary Figures 1–4**). Separate subgroup analyses were conducted to examine whether the overall results were robust and to explore the source of potential heterogeneity.

### Data analysis

We calculated pooled RRs for short-, mid- and long-term all-cause mortality (with their 95% confidence intervals) associated diabetes for each sex, using an inverse-variance weighting version of the DerSimonian and Laird random-effects models provided by Cochrane Review Manager (RevMan) software, version 5.4 (24). The estimated RRs from each study were treated as one stratum, and stratum-specific RRs were pooled together to create an overall estimate of RRs by taking a weighted average of the stratum-specific RRs (18). Studies with more individuals contributed more to the pooled RRs than did smaller studies (18). RevMan 5 used the following formula to calculate risk ratio of all-cause mortality for each study (25): (i=study ID, a=all-cause mortality rate in diabetes patients, n1=total number of diabetes patients; c=all-cause mortality rate in non-diabetes patients, n2=total number of non-diabetes patients)


$$RR_{i}=\frac{a_{i}/n_{1i}}{c_{i}/n_{2i}}$$

The standard error of the log risk ratio was calculated using the following formula (25):


$$SE\left\{\text{ln }\left(RR_{i}\right)\right\}=\sqrt{\frac{1}{a_{i}}+\frac{1}{c_{i}}-\frac{1}{n_{1i}}-\frac{1}{n_{2i}}}$$

The data from included studies were used to calculate sex-specific RRs comparing adults with diabetes vs. adults without diabetes. Our main analyses included only studies that provided adjusted RRs to describe the independent association between diabetes and all-cause mortality after AMI. We also performed analysis to determine unadjusted RRs for short-, mid-, and long-term all-cause mortality after AMI (patients with diabetes vs. without diabetes), using raw numbers of death and total participants at risk for death specific to each sex and diabetes status. For studies that reported all-cause mortality and other endpoints (e.g., cardiovascular death, the incidence of recurrent AMI), only all-cause mortality was used in the statistical analysis.

Because diagnostics and treatment for diabetes and AMI have changed considerably over time, we assessed specific time trends extant in pooled sex-specific RRs for mortality by plotting the adjusted RRs against the mid-point of the study period. A 2-sided P < 0.5 was considered significant for all analyses. All statistical analysis was performed using RevMan (version 5.4, Cochrane collaboration, Oxford, UK) and R (version 3.3.2).

### Sensitivity and subgroup analysis

Meta-analyses were done on 19 observational studies. Because only one randomized control trial met our inclusion criteria and provided unadjusted RRs only, we performed sensitivity analysis by including the one randomized control trial to the observational studies that also provided unadjusted RRs to see if the unadjusted pooled RRs for each sex were consistent (**Supplementary Table 1**). We also performed subgroup analyses to examine crucial factors that might affect sex-specific all-cause mortality and assess sources of heterogeneity. Subgroup analyses included stratification by patients’ mean age as reported by each study (< 65 years and ≥65 years) and midpoint of the study period (Studies before 2008 and studies from 2008). These subgroup cut-off points were determined by evidence showing a higher mortality among AMI patients aged ≥65 years, and that CVD outcome trials are required to prove CV risk profiles in antihyperglycemic agents by the FDA beginning in 2008 (16, 26).

## Results

The initial search yielded 4,212 articles (**Figure 1**), of which 565 were duplicates. Titles and abstracts were screened for the 3647 articles [QD]. Of these, 215 articles met the inclusion criteria and underwent full text review, and 20 articles were found to have provided enough information to perform the analysis [QD & EW]. The 20 included articles reported sex-specific death rates after AMI in people with and without diabetes from 13 countries (**Table 1**). Nineteen studies were retrospective and prospective observational cohort studies established by linking AMI/hypertension registries or administrative data. One study analyzed data of patients enrolled in a randomized controlled trial (41).

**Table 1:** Basic characteristics of included studies.

Source	Source of sample (Country)	Study enrollment period	Follow-up Period	Total No. of participants	No. of women with diabetes	No. of men with diabetes	No. of deaths (women with diabetes)	No. of deaths (men with diabetes)	Age in years of women with diabetes (SD) or age range	Age in years of men with diabetes (SD) or age range	Comparison group
**Abbott et al., 1988** (5)	The Framingham Study (USA)	1948-1982	34 Years	609	37	55	9	10	69.4 (8.4)	62.2 (9.8)	Patients with and without a diabetes diagnosis before an initial MI occurred
**Abbud et al., 1995** (27)	Myocardial infarction Acquisition System (USA)	1986-1987	3 years	37921	3503	7166	2200	2368	Range (30-89)	Range (30-89)	Diabetic and non-diabetic patients with an acute MI diagnosis discharged from 90 nonfederal hospitals in 1986 and 1987
**Ahmadi et al., 2015** (28)	Myocardial infarction registry of Iran’s Cardiovascular Diseases Surveillance System (Iran)	2012 -2013	1 year	20750	1911	2701	193	295	61.9 (11.7)	60.9 (13.8)	Diabetic and non-diabetic patients hospitalized with a new presentation with MI (across 540 hospitals) between April 2012 to March 2013
**Behar et al., 1997** (29)	SPRINT study (Israel)	1981- 1983	10 years	5255	482	766	41	44	64.5 (9)	62 (10)	Patients with diabetes after an acute MI and patients without diabetes after an acute MI
**Blondal et al., 2012** (6)	EMIR, EHIF and EPR database (Estonia)	2006-2009	2.7 years	1652	142	155	26	25	69.3 (9.6)	65 (9.4)	Diabetic and non-diabetic patients with AMI who have undergone percutaneous coronary intervention (PCI)
**Brophy et al., 2010** (30)	National hospital admission datasets for England and Wales	2003-2006	1 year	157142	26213	41829	4737	5318	70*	70	Diabetic and non-diabetic patients hospitalized for MI using all hospital admissions in England and Wales for the years 2003-2006
**Chun et al., 1997** (31)	WHO MONICA Project (Australia)	1985-1994	28 days after onset of AMI	5322	224	333	32	44	63 (10)	61 (12)	MI patients with and without diabetes aged 30-69 years from the lower Hunter Region of New South Wales, Australia
**Crowley et al., 2003** (32)	Worcester Heart Attack Study (USA)	1975-1999	In-hospital mortality	10057	1280	1354	61	67	72.2	67.3	Diabetic and non-diabetic patients with confirmed AMI from the Worcester metropolitan area
**Gore et al., 2012** (33)	National Registry of Myocardial Infarction (USA)	1994-2006	In-hospital mortality	466972	928276	1080984	2782	3753	Median age for cohorts 1994 (69.7); 1998 (70.7); 2000 (72.0); 2006 (71.0)	Median age for cohorts 1994 (69.7); 1998 (70.7); 2000 (72.0); 2006 (71.0)	MI patient with and without diabetes data obtained from the NRMI treated in 1964 hospitals
**Greenland et al., 1991** (34)	SPRINT Registry (Israel)	1981-1983	1 year	5839	443	1552	35	114	67.5	61.4	Acute MI patients with and without diabetes from 14 different hospitals throughout Israel
**Hu et al., 2005** (7)	Finnish cohort studies (Finland)	1972, 1977, 1987,1992 and 1997	12 years	1454	47	99	17	38	58.7	60.1	Diabetic and non-diabetic patients with MI at baseline
**Koek et al., 2007** (35)	linked national registers in Netherlands	1995-2000	5 years	21565	3147	2907	1214	975	68.7 (11)	73.2 (10.3)	Patients with and without diabetes hospitalized for first acute MI in 1995
**Lopez-de-Andres et al., 2021** (36)	Spanish National Hospital Discharge Database	2016, 2017, 2018	In-hospital mortality	154348	15220	21802	1692	2004	75.87 (11.43)	67.33 (11.88)	Diabetic and non-diabetic patients aged 40 years or over collected by the SNHDD in years 2016, 2017, and 2018
**Meisinger et al., 2010** (9)	The MONICA/KORA Myocardial infarction registry (Germany)	1998- 2003	4.3 years	2443	370	964	84	168	61.8 (8.6)	64.7 (8)	Patients with and without DM consecutively hospitalized with a first-ever AMI from January 1998 to December 2003 recruited from a population-based MI registry (MONICA/KORA)
**Miettinen et al., 1998** (12)	FINMONICA Myocardial Infarction Register (Finland)	1988-1992	1 year	4062	366	874	83	178	58.2 (6.9)	55.8 (7.5)	Diabetic and non-diabetic patients residing in three geographically defined study areas who were in the age-group 25-64 years, and either were hospitalized because of a suspected MI
**Mukamal 2001** (37)	Determinants of Myocardial Infarction Onset Study (USA)	1989-1993	3.7 years	1935	164	235	39	55	65 ± 11*	65 ± 11	Diabetic and non-diabetic patients sustaining an AMI
**Muller et al., 2004** (38)	Patient from The Heart Center Bad Krozingen (Germany)	1996-1999	5 years	1433	83	187	5	30	68 ± 9*	68 ± 9	Diabetic and non-diabetic patients with unstable angina and non-ST-segment elevation MI
**Norhammar et al., 2003** (39)	Register of RIKS-HIA (Sweden)	1995-1998	1 years	25633	1869	3323	81	104	70	70	Diabetic and non-diabetic patients below the age of 80 years from the Register of Information and Knowledge about Swedish Heart Intensive Care Admissions (RIKS-HIA)
**Schramm et al., 2008** (40)	Danish Civil Registration System (Denmark)	1997-2002	5 years	79574	2269	4150	1383	1454	72.1 (11.7)	66.5 (11.5)	Diabetes patients receiving glucose-lowering medications and nondiabetics with and without a prior MI.
**Zuanetti et al., 1993** (41)	GISSI-2 study (Italy)	1988-1989	6 months	11667	1144	2532	145	75	NA	NA	Non-diabetic patients and insulin-dependent and noninsulin-dependent diabetic patients in the GISSI-2 study

NA, not available; AMI, acute myocardial infarction; DM, diabetes mellitus; SNHDD, Spanish National Hospital Discharge Database; GISSI-2, The second trial conducted by the Gruppo Italiano per lo Studio della Sopravvivenza nell’Infarto Miocardico (**GISSI**) (Italian group for the study of the survival of myocardial infarction); PCI, percutaneous coronary intervention; NRMI, National Registry of Myocardial Infarction.

Included studies were published between 1988 and 2021, with the cohort’s enrollment period ranging from 1945 to 2018. The sample size ranged between 609 (AMI survivors in the Framingham study, USA) (5) to 1,734,432 (National Registry of Myocardial Infarction, USA) (33). Ten studies reported sex-specific mean age for enrolled patients with diabetes, with the average age of men with diabetes ranging between 55.8, SD (7.5) (12) and 70.9, SD (11) (36), and women with diabetes ranging between 58.2, SD (6.9) (12) and 76.9, SD (10) (36). The follow-up period of the cohort varied across the included studies (ranging from in-hospital to 34 years after discharge for AMI hospitalization). Additional clinical and methodological characteristics of the included studies were listed in **Supplementary Table 2**.

### Quality of the studies

The Revised Cochrane risk-of-bias tool for randomized trials (RoB2) (22) and National Heart, Lung, and Blood Institute’s Quality Assessment Tool for Observational Cohort and Cross-Sectional Studies (20) were used to assess quality of the included articles. Most of the reviewed studies demonstrated good overall quality, and reported sufficient details related to study objective, study population, outcomes and exposure measures, timeframe, and key potential confounding variables (**Supplementary document for ROB2 & NHLBI tool**). However, few studies have provided reasons for non-participation at each stage or explained how missing data were addressed. Six studies provided unadjusted estimates only (9, 27, 32, 36, 39, 41). Of the 14 studies that included confounder-adjusted estimates, 2 did not clearly indicate which confounders were adjusted for (5, 28). Only 1 of the 14 studies also published unadjusted estimates (35).

### Pooled sex-specific RRs for short-term mortality associated with diabetes after AMI

#### Summary of RRs of short-term mortality for men

**Figure 2:**
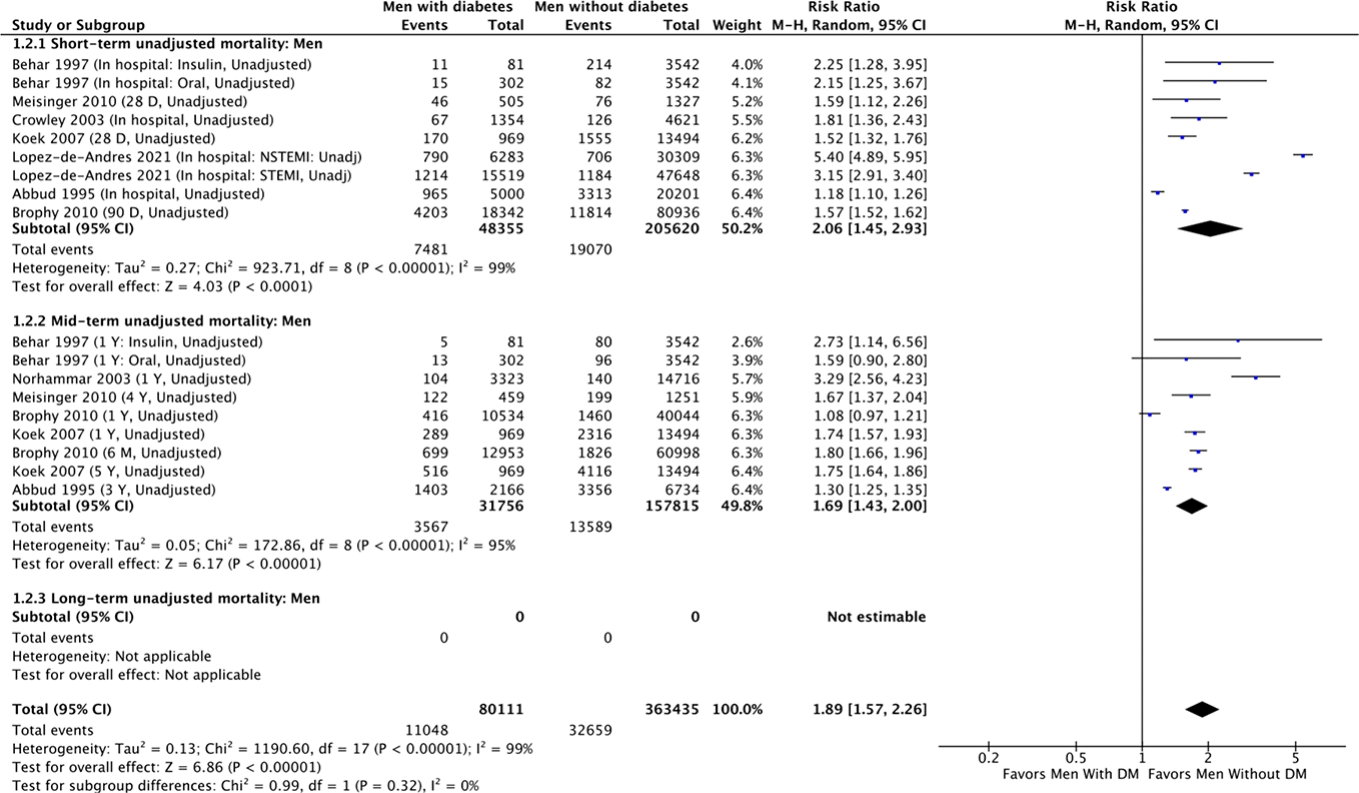
Summary of unadjusted short-term, mid-term and long-term risk estimates for post-AMI all-cause mortality in men with diabetes compared to men without diabetes. (Data extracted from 8 studies and including of total of 80,111 men with diabetes and 363, 435 men without diabetes).

Seven cohort studies reported unadjusted short-term all-cause mortality comparing men with diabetes vs men without diabetes (9, 27, 29, 30, 32, 35, 36), and involved 48,355 men with diabetes and 205,620 men without diabetes. There were 7481 (15.5%) and 19070 (9.3%) short-term deaths in men with and without diabetes, respectively (**Figure 2**). In the unadjusted analysis, men with diabetes had a significantly higher risk of short-term all-cause mortality compared with men without diabetes (RR, 2.06; 95% CI, 1.45-2.93 [P<0.0001, *I* ^2 ^= 99%]). With the one randomized controlled trial included in the sensitivity analysis, the strength of association for men with diabetes remained robust (**Supplementary Figure 5**).

**Figure 3:**
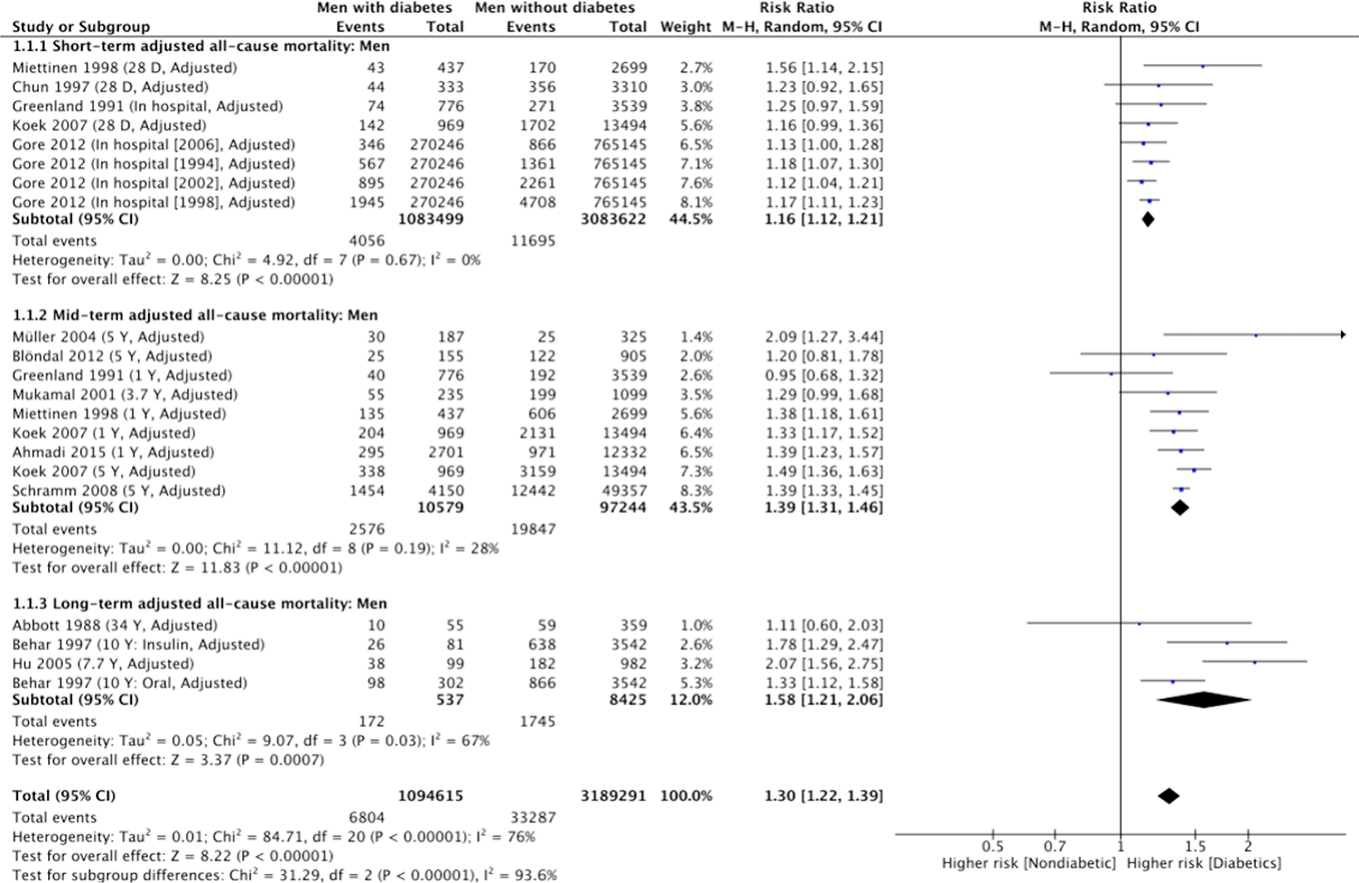
Summary of adjusted short-term, mid-term and long-term risk estimates for post-AMI all-cause mortality in men with diabetes compared to men without diabetes. (Data extracted from 13 studies and including of total of 1, 094, 615 men with diabetes and 3, 189, 291 men without diabetes).

However, using adjusted RRs from 5 cohort studies, the strength of association for short-term mortality in men with diabetes compared with men without diabetes significantly attenuated, though it remained significant (RR, 1.16; 95% CI, 1.12-1.20 [P<0.001, *I* ^2 ^= 0%) (**Figure 3**) (12, 31, 33–35). All 5 included cohort studies adjusted for age in their analysis (**Supplementary Table 3**). There was no significant heterogeneity in the effect between studies that reported the individual adjusted RRs.

#### Summary of RRs of short-term mortality for women

**Figure 4:**
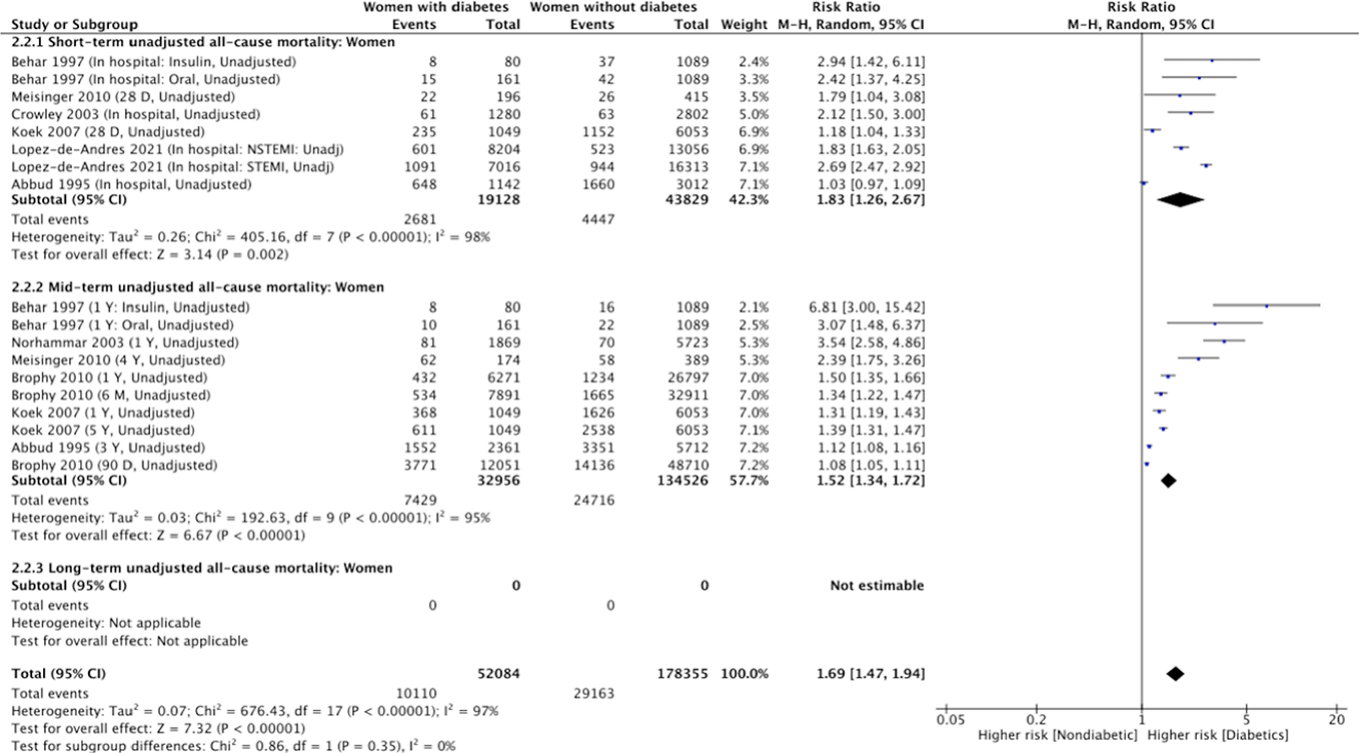
Summary of unadjusted short-term, mid-term and long-term risk estimates for post- AMI all-cause mortality in women with diabetes compared to women without diabetes. (Data extracted from 8 studies and including of total of 52, 084 women with diabetes and 178, 355 women without diabetes).

Six studies involving 19,128 women with diabetes and 43,829 women without diabetes (62,957 patients total) reported unadjusted short-term all-cause mortality numbers for women with and without diabetes (9, 27, 29, 32, 35, 36). They found 2681 (14%) and 4447 (10%) deaths at short-term in women with and without diabetes, respectively (**Figure 4**). Women with diabetes (vs. women without diabetes) had significantly higher risk of short-term all-cause mortality in the unadjusted analysis (RR, 1.83; 95% CI, 1.26-2.67). This was true in the sensitivity analysis including the randomized control trial (RR, 1.57; 95% CI, 1.39-1.77 [P<0.001; *I* ^2 ^= 95%] (**Supplementary Figure 6**). There was significant heterogeneity among the six cohort studies (**Table 2b** and **Supplementary Figure 4**, χ2 = 405.1; 7d.f.; P<0.0001; *I* ^2 ^= 98%), and no evidence of publication bias through visual inspection of funnel plots for unadjusted short-term mortality (**Supplementary Figure 1**). Besides geographical location differences across the six studies, the high heterogeneity may also relate to the type of AMI (first AMI versus prior AMI) and diabetes definition differences (type 2 diabetes per ICD9 codes versus unspecified diabetes type) (**Supplementary Table 2**).

**Figure 5:**
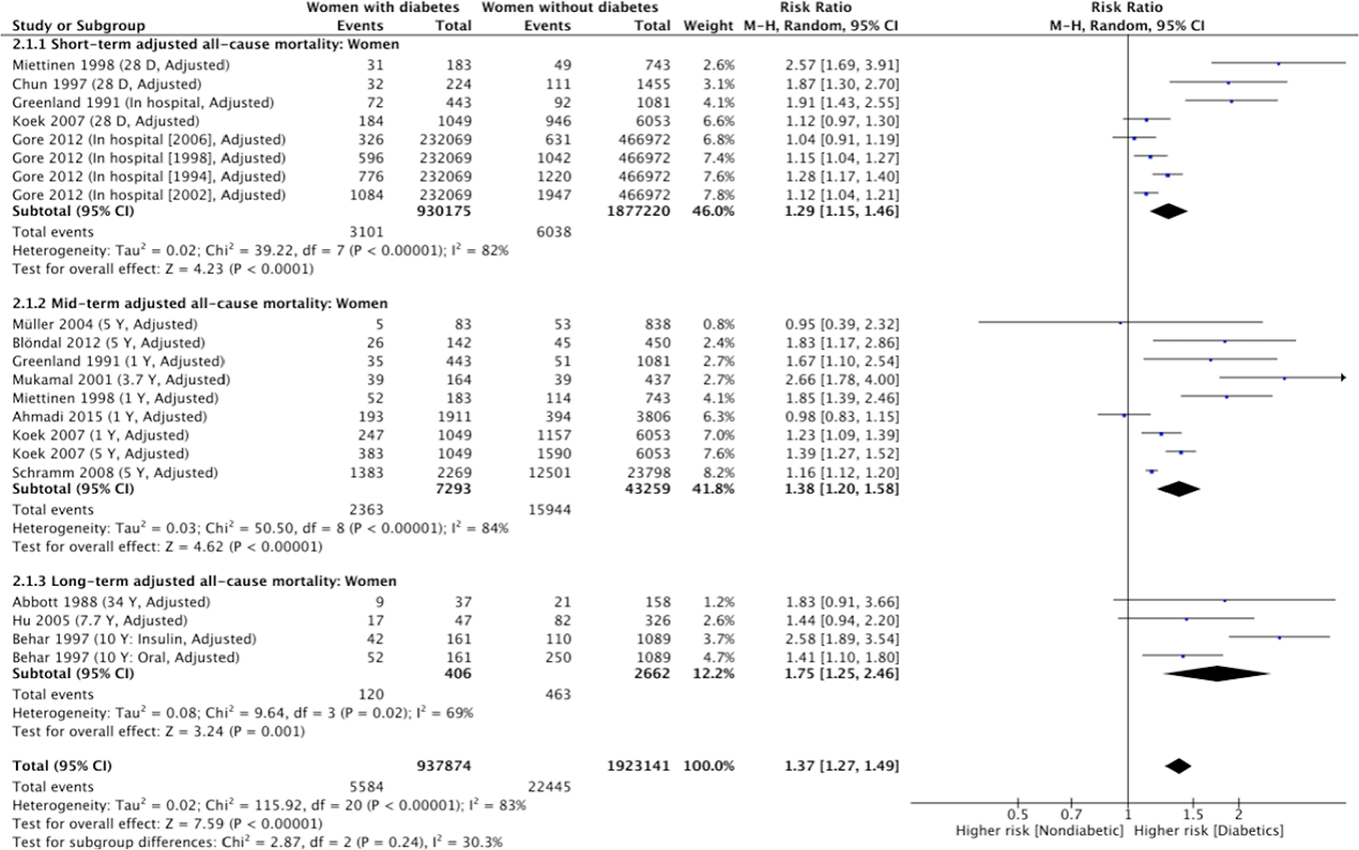
Summary of adjusted short-term, mid-term and long-term risk estimates for post-AMI all-cause mortality in women with diabetes compared to women without diabetes. (Data extracted from 13 studies and including of total of 937,874 women with diabetes and 1, 923,141 women without diabetes).

In the adjusted analysis (**Figure 5**), the summary RR of short-term mortality was 1.29 (95% CI: 1.15-1.46) in women with diabetes compared with women without diabetes (12, 31, 33–35). There was considerable between-study heterogeneity in the effect of diabetes (**Figure 5**, χ2 = 39.27, 7d.f.; P<0.0001; *I* ^2 ^= 82%). The significant heterogeneity was largely due to the studies by Gore et al. (33) and Koek et al. (35), both of which defined diabetes using medical records, included larger sample sizes, and had adjusted for demographics and CVD risk factors such as prior CVD history in their analyses. After excluding these two studies, the summary RR of short-term mortality for women increased to 2.03 (95% CI: 1.66-2.48), and no heterogeneity was present between studies (χ2 = 1.57, 2 d.f.; P = 0.46, *I* ^2 ^= 0%) (**Supplementary Figure 7**).

#### Sex differences in short-term mortality associated with diabetes

Based on the sex-specific analyses, the summary RR of adjusted short-term mortality after AMI due to diabetes was slightly higher for women than for men. However, this sex difference associated with diabetes was not observed in the pooled analysis based on the unadjusted data from the cohort studies.

### Pooled sex-specific RRs for mid-term mortality associated with diabetes after AMI

#### Summary of RRs of mid-term mortality for men

Six studies reported unadjusted mid-term mortality comparing men with diabetes to men without diabetes (9, 27, 29, 30, 35, 39), and involved 31,756 men with diabetes and 157,815 men without diabetes (**Figure 2**). The mid-term deaths in men with diabetes were 3567 (11% rate) and 13,589 in men without diabetes (8.6% rate). In the unadjusted analysis of mid-term mortality, men with diabetes had increased risk of all-cause mortality compared with men without diabetes (RR, 1.69; 95% CI 1.43-2.00 [P<0.001, *I* ^2 ^= 95%]). The inclusion of the randomized control trial into the cohort studies did not change the conclusion (**Supplementary Figure 5**) (RR, 1.73; 95% CI 1.48-2.04; [P<0.0001, *I* ^2 ^= 94%]).

In the adjusted analysis, using adjusted RRs from 8 studies (6, 12, 28, 34, 35, 37, 38, 40), the strength of association for mid-term all-cause mortality in men with diabetes (compared with men without diabetes) was attenuated but remained significant (**Table 2a**. RR, 1.39; 95% CI, 1.31-1.46 [P<0.001, *I* ^2^ = 28%]). Heterogeneity between cohort studies was further reduced (*I* ^2^ = 25%) after excluding the two studies that showed a possibly higher influence on the effect estimate (**Supplementary Figure 8**).

**Table 2:** Pooled sex-specific risk ratio (RR) for post-AMI all-cause mortality associated with diabetes.

2a. Pooled RRs for Men with Diabetes Compared with Men without Diabetes.					
Adjustment type	Number of studies	Association effect (95% CI)	*P* for effect	Heterogeneity	*P* for heterogeneity
Short-term unadjusted	7	2.06 (1.45-2.93)	<0.0001	99%	<0.00001
Short-term unadjusted (RCT included)*	8	2.20 (1.59-3.04)	<0.00001	99%	<0.00001
Short-term adjusted	5	1.16 (1.12-1.20)	<0.00001	0%	0.64
Mid-term unadjusted	6	1.69 (1.43-2.00)	<0.0001	95%	<0.00001
Mid-term unadjusted (RCT included)*	7	1.73 (1.48-2.04)	<0.0001	94%	<0.00001
Mid-term adjusted	8	1.39 (1.31-1.46)	<0.0001	28%	<0.20
Long-term unadjusted	0	NA	NA	NA	NA
Long-term adjusted	4	1.58 (1.22-2.05)	0.0006	66%	0.03
2b. Pooled RRs for Women with Diabetes Compared with Women without Diabetes.					
Adjustment type	Number of studies	Association effect (95% CI)	*P* for effect	Heterogeneity	*P* for heterogeneity
Short-term unadjusted	6	1.83 (1.26-2.67)	0.002	98%	<0.0001
Short-term unadjusted (RCT included)*	7	1.72 [1.25, 2.38]	<0.00001	95%	<0.00001
Short-term adjusted	5	1.29 (1.15-1.46)	<0.0001	82%	<0.0001
Mid-term unadjusted	7	1.52 (1.34-1.72)	<0.0001	95%	<0.00001
Mid-term unadjusted (RCT included)*	8	1.57 (1.39-1.77)	<0.0001	95%	<0.00001
Mid-term adjusted	8	1.38 (1.20-1.58)	<0.0001	84%	<0.00001
Long-term unadjusted	0	NA	NA	NA	NA
Long-term adjusted	3	1.76 (1.25-2.47)	0.001	69%	0.02

RR, risk ratios.

AMI, acute myocardial infarction.

RCT, randomized controlled trial.

NA, information not available due to lack of data from included studies.

*The data extracted from the one randomized control trial was included as a sensitivity analysis. This study paper only reported raw short- and mid-term all-cause mortality associated with diabetes.

#### Summary of RRs of mid-term mortality for women

Seven studies involving 32,956 women with diabetes and 134,526 women without diabetes (167,482 patients total) provided unadjusted mid-term mortality for women with and without diabetes (**Figure 4**). They found 7,429 (22.5% rate) and 24,716 (18.4% rate) deaths at mid-term follow-up in women with and without diabetes, respectively. In the unadjusted analysis, women with diabetes had significantly higher risk of mid-term all-cause mortality compared with women without diabetes (**Table 2b**, RR, 1.52; 95% CI, 1.34-1.72 [P<0.0001, *I* ^2 ^= 95%]). In the sensitivity analysis including data from the randomized control trial, the association of women with diabetes (vs. women without diabetes) and increased mid-term mortality remained significant (**Supplementary Figure 6**).

However, in the adjusted analysis using adjusted RRs from 8 studies, the strength of association for mid-term mortality in women with diabetes compared to women without diabetes attenuated but remained significant (RR, 1.38; 95% CI, 1.20-1.58 [P<0.0001, *I* ^2 ^= 84%]) (**Figure 5**). Heterogeneity between studies was not altered much after removal of studies possibly influenced most on the pooled risk estimates (*I* ^2 ^= 85%) (**Supplementary Figure 7**). Assessment of these eight studies’ clinical and methodological characteristics noted data source (population cohort vs. AMI patient from a single heart center) and diabetes definition differences might explain the high heterogeneity between studies (**Table 1** and **Supplementary Table 2**).

#### Sex differences in mid-term mortality associated with diabetes

Although the summary RRs for mid-term all-cause mortality for men was slightly higher than those for women based on unadjusted data from cohort studies (1.69 vs. 1.52), there was little difference between the adjusted summary RRs for mid-term all-cause mortality due to diabetes between women and men (1.39 vs. 1.38).

### Pooled sex-specific RRs for long-term mortality associated with diabetes after AMI

#### Summary of RRs of long-term mortality for men

No studies with a follow-up period >5 years reported an unadjusted risk estimate or crude rates of long-term all-cause mortality for men with and without diabetes (**Figure 2**). Based on 3 cohort studies providing adjusted RRs (5, 7, 29), the adjusted summary RRs for long-term all-cause mortality after AMI among men with diabetes versus men without was 1.58 (95% CI 1.22-2.05) (**Table 2**). There was substantial heterogeneity among the 3 studies (**Figure 3**, χ2 = 8.82; 3d.f.; P=0.03; *I* ^2 ^= 66%), which indicates extreme inconsistency in the adjusted long-term effect of diabetes in men between the studies. Heterogeneity changed to moderate after removing a study by Behar and colleagues (29) influenced significantly on the effect estimate (χ2 = 3.29; 2d.f.; P=0.19; *I* ^2 ^= 39%) (**Supplementary Figure 8**). While all 3 studies adjusted for clinically related covariates such as CVD risk factors in addition to demographic characteristics, the study led by Behar (29) also adjusted for AMI related characteristics such as the site of myocardial infarction in their analyses (**Supplementary Table 3**).

#### Summary of RRs of long-term mortality for women

Summary of unadjusted RRs of long-term all-cause mortality comparing women with diabetes and women without diabetes was also unavailable due to lack of extracted data (**Figure 4**). In the adjusted analysis (**Figure 5**), using adjusted RRs reported by 3 studies (5, 7, 29), the association of women with diabetes (vs. women without diabetes) and an increased risk for long-term all-cause mortality was significant (RR, 1.76; 95% CI 1.25-2.47; P=0.01; *I* ^2 ^= 69%). Heterogeneity between studies was reduced to *I* ^2 ^= 0 after excluding the study led by Behar that possibly contributed most to between-study heterogeneity (**Supplementary Figure 7**).

#### Sex differences in long-term mortality associated with diabetes

Based on 3 observational cohort studies that reported adjusted RRs, the sex-specific summary of long-term mortality associated with diabetes was somewhat higher in women than in men (1.76 vs. 1.58).

### Subgroup analysis and time trends

#### Midpoint of study period

In the subgroup of studies with a midpoint of study period from 2008, only 2 out of the 19 cohort studies reported adjusted RRs or crude rates of all-cause mortality for the subgroup analysis in men and women (6, 28). Based on these 2 cohort studies with a mid-term follow-up, the pooled adjusted estimates of all-cause mortality were relatively consistent when stratified by midpoint of the study period (Studies before 2008 and studies from 2008) and were in the same direction as those from the main analyses (**Supplementary Figures 9-10**). In the subgroup of studies before 2008, the pooled adjusted RRs of long-term all-cause mortality were significantly higher than RRs of short-and mid-term all-cause mortality for men, indicating that the effect of diabetes on risk of all-cause mortality tended to increase with the duration of follow-up in men (**Supplementary Figure 10**, subgroup difference: p<0.0001). However, in women, duration of follow-up period after AMI was not associated with increased risk of all-cause mortality due to diabetes as the pooled adjusted RRs of short-, mid- and long-term all-cause mortality were not statistically different in women (**Supplementary Figure 9**, subgroup difference: p=0.19).

#### Age

We further divided men and women participants between those younger than 65 years and 65 years or older. In men and women, both age groups favored individuals without diabetes. Short-term all-cause mortality was higher in women younger than 65 years (RR, 2.15; 95% CI 1.58-2.94; P<0.0001; *I* ^2 ^= 21%) when compared with women 65-years or older (RR, 1.19; 95% CI 1.08-1.31; P=0.0004; *I* ^2 ^= 75%) (**Supplementary Figure 11**). This was true in the subgroup analysis restricted to adjusted data from men as well (<65 years: RR, 1.32; 95%CI 1.12-1.55; P=0.0008; *I* ^2 ^= 0%; 65-years or older: RR 1.15; 95%CI 1.11-1.20; P<0.00001; *I* ^2 ^= 0%) (**Supplementary Figure 12**). However, the risk of mid-term mortality was slightly higher in those 65-years or older when compared with those younger than 65 years in both men and women. Only one study reported long-term mortality risk associated with diabetes for men and women 65-years or older (5). The summary adjusted RRs for long-term all-cause mortality appeared slightly higher in women 65-years or older than RRs in women younger than 65 (1.83 vs. 1.74) (**Supplementary Figure 11**). Overall, pooled RRs of all-cause mortality associated with diabetes differed significantly between age groups in men (subgroup difference: P<0.0001) and women (subgroup difference: P=0.004).

#### Time trends

**Figure 6:**
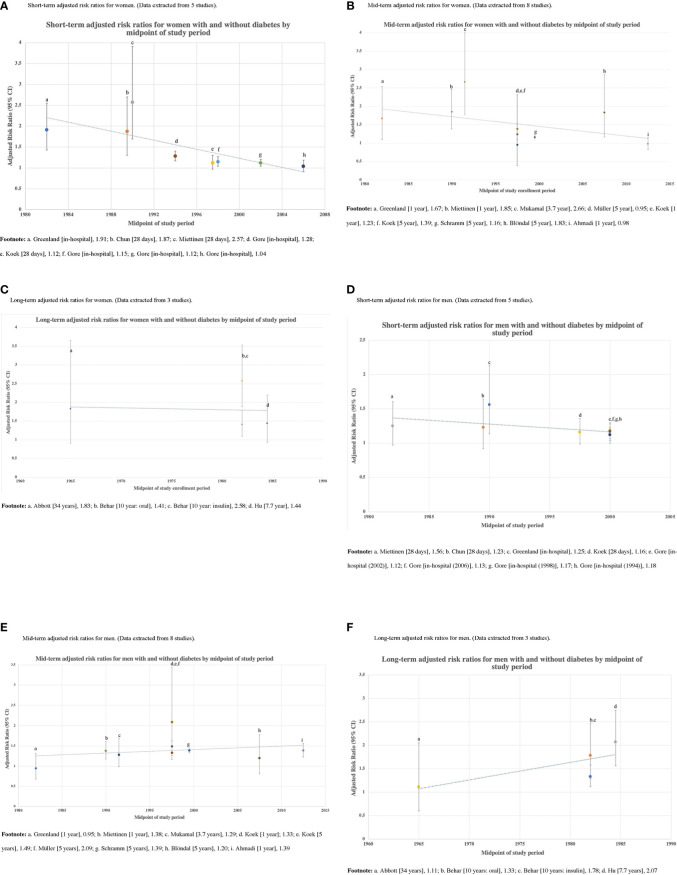
Time trends in adjusted short-, mid-, and long-term risk ratios for women and men with and without diabetes by the midpoint of study period.

Study enrollment periods varied in length from in-hospital to 34 years, and data collected by these studies spanned the years of 1988 to 2021. We evaluated whether the reported adjusted short-, mid- and long-term all-cause mortality RRs associated with diabetes for men and women changed over time (**Figures 6A–F**). We found a significant downward trend for adjusted short-term and mid-term all-cause mortality in women, suggesting the all-cause mortality gap between women AMI patients with and without diabetes narrowed significantly over time. Among men AMI survivors, a downward trend was noted only for short-term all-cause mortality.

## Discussion

### Summary of main findings

We conducted an up-to-date and comprehensive systematic review and meta-analysis of sex-specific post-AMI all-cause mortality associated with diabetes based on 19 observational and 1 randomized control trial studies published between the years of 1988 and 2021. We evaluated sex-specific risk of all-cause post-AMI mortality associated with diabetes by different duration of follow-up period (short-, mid-, long-term) and analyzed summary estimates of risk using both raw and adjusted data. The main findings include the following: (1) Diabetes was significantly and independently associated with short-term, mid-term and long-term post-AMI all-cause mortality in both men and women, (2) While the risk of all-cause mortality associated with diabetes increased with longer follow-up in men, the risk in women remained the same across different follow-up periods. Women with diabetes had an almost 1.5-fold increase in risk of all-cause mortality at short-, mid- and long-term follow-up, compared with women without diabetes, (3) Although the effect of diabetes on mid-term all-cause mortality was similarly significant in both sexes, the RRs of all-cause mortality associated with diabetes appeared to be greater for women than for men at short- (in-hospital or within 90 days after discharge) and long-term (>5 years) follow-up periods using the adjusted data.

Compared with previous meta-analyses on all-cause mortality risk associated with diabetes in men and women (13, 14, 42), the main strength of this meta-analysis is pooling the unadjusted and adjusted sex-specific RRs for post-AMI all-cause mortality from each study separately and analyzing separately for short-, mid- and long-term follow-ups. Two previous meta-analyses have included studies that did not adjust for CVD risk factors (14, 42). Also, none of the earlier meta-analyses provided evidence of potential difference in the effect of diabetes on short-, mid-, or long-term all-cause mortality risk after AMI in men or women (13, 14, 42). The differences in RRs for post-AMI all-cause mortality between people with and without diabetes were attenuated in both sexes after adjustments for covariates, suggesting the presence of confounding factors that might partly explain the observed excess mortality for men and women with diabetes (14). Another explanation could be that proven cardiac interventions remain underused in adults with diabetes, partly due to delayed presentation and more atypical symptoms onset of AMI (39, 43). Since most studies included in our meta-analyses could not account for potential confounding variables, further research is needed to evaluate whether adjustment for known CVD risk factors, AMI characteristics, and AMI/diabetes treatment might eliminate the remaining mortality disparity between patients with and without diabetes. Future systematic reviews may update the present meta-analysis when more evidence has accumulated for potential AMI/diabetes-related factors influencing the impact of diabetes on post-AMI all-cause mortality in women and men. These factors include but are not limited to AMI or diabetes type, AMI interventions received during hospitalization, type of diabetes medication used, and duration of diabetes. Our findings should help improve awareness of the increased post-AMI all-cause mortality risk in both men and women with diabetes and encourage a more diligent use of proven treatment approaches for patients with diabetes and AMI.

Considerable evidence supports the fact that the presence of diabetes reduces the protective effect of female sex on coronary heart disease risk (7, 44–46). The U.S. population data also showed disparities in all-cause and CVD mortality rates between women with and without diabetes (47). It has been reported that women with diabetes are more likely to be older, obese, have a higher prevalence of hypertension, dyslipidemia, and greater waist-to-hip ratio than women without diabetes (44, 48, 49). Certain CVD risk factors, such as endothelial function, have also been shown to be impaired to a greater extent in premenopausal women with diabetes than men with diabetes (50). Non-traditional risk factors such as glucose variability and poverty may also be important in women with diabetes even though very few studies have examined the effect of non-traditional risk factors in diabetes independent of traditional risk factors (2). Although only one study included in this meta-analysis provided post-AMI long-term all-cause mortality for women 65-years or older, our findings support a more deleterious impact of diabetes on mid-term all-cause mortality in older women compared to women younger than 65 years. Age itself is a significant risk factor for CVD for women and men (46). Among women with diabetes aged over 65, older age and menopause might additively influence the elevated probability of mortality after AMI (51, 52). Menopause is accompanied with estrogen withdrawal and can trigger molecular or functional changes in calcium homeostasis related to proteins in cardiomyocytes (52). Reduction in estrogen may also be associated with impaired myocardial function and structure (53). Since our study did not have data on estrogen levels or menopausal status, future studies with a longer follow-up period are needed to test if menopause influences the association between diabetes and post-AMI all-cause mortality in women.

Although this analysis did not pool post-AMI all-cause mortality RRs from a direct comparison of women with diabetes to men with diabetes, the sex-specific adjusted RRs of all-cause mortality associated with diabetes were slightly higher for women than for men at both short- and long-term follow-up. However, in our study, the sex differences in adjusted summary RRs for mid-term all-cause mortality were not pronounced. Our findings suggest that the association between diabetes and risk of all-cause mortality might differ by duration of follow-up after AMI. Sex differences in RRs of post-AMI all-cause mortality associated with diabetes have been repeatedly reported (14, 42). Most studies that have demonstrated a persistent mortality risk difference associated with diabetes between men and women did not account for conventional risk factors (e.g. CVD risk factors), and mechanisms underlying sex differences are poorly understood (14). Two studies included in our review suggest that women with diabetes have a longer delay between symptom onset and AMI admission than men with diabetes (6, 12). Studies also showed that women with diabetes were less likely to have received aspirin or reperfusion therapy within 12 hours after symptom onset and were less likely to undergo cardiac catheterization and coronary angioplasty than men with diabetes (6, 32). Additionally, the occurrence of in-hospital complications, especially cardiogenic shock and heart failure following AMI, were significantly higher in women with diabetes (29, 32, 34, 41). Our study cannot determine whether sex differences in diabetes-associated death are due to the biological effect of diabetes in women or is the socio-cultural effect of women with diabetes being cared for by the health system. Still, our results highlight the importance of a sex-specific approach in providing AMI care in acute settings.

### Limitations

Several limitations should be considered when interpreting the results. First, we are limited to variables measured and end points published in each study, as with many meta-analyses. Also, many recently published studies related to the topic did not provide sex-specific post-AMI all-cause mortality in both patients with and without diabetes, thus they can’t be included in the review (54–56). Although we followed the PRISMA guidelines and used an extensive search strategy to identify existing literature in multiple databases, the majority of the included studies were observational cohort studies with greater potential for bias and between-study heterogeneity than randomized controlled trials (14). We explored the causes of the heterogeneity between studies for post-AMI all-cause mortality through subgroup analyses and assessment of study quality and clinical and methodological characteristics of included studies. The between-study heterogeneity in women remained high throughout subgroup analyses by participants’ average age and mid-point of the study period. Nevertheless, our assessment of the study’s clinical and methodological characteristics suggests that the greater heterogeneity could result from differences in participants’ characteristics (population-based study versus AMI registry); AMI types (first AMI or recurrent AMI); diabetes definition/type, and in-hospital interventions received by participants. The greater heterogeneity could also represent actual variation in the effect of diabetes in post-AMI all-cause mortality in women. Additionally, it may be related to differences in care provided to women across different studies, which may be more variable than in men, given historically documented examples of sex biases within healthcare.

Another limitation was the absence of diabetes-related data, which has been shown to affect mortality risk (e.g., type of diabetes was not reported in many studies (14), duration of diabetes (45, 57), glycemic control (58), diabetes medication type (16), and diabetes complications (59)). Two included studies have distinguished participants with insulin-dependent diabetes from those with non-insulin dependent diabetes (29, 41), but rarely all diabetes-related factors are measured, and whether these unmeasured factors could account for the excess risk associated with diabetes after AMI is unclear. Further, few studies accounted for the types of percutaneous coronary intervention (PCI) in exploring the impact of diabetes on AMI mortality. Given the lessons learned from FREEDOM, BARI 2D and COURAGE trials, future studies should evaluate the role of PCI types in the association between diabetes and survival in men and women (60). Additionally, the literature search was conducted in two databases only and was restricted to English language articles. Using multiple databases and including non-English studies could help find more results and ensure that all relevant studies are identified.

Finally, our meta-analysis does not account for the sex-specific effects of undiagnosed diabetes at baseline or newly developed diabetes during follow-up. The diagnosis of diabetes in most studies was based solely on medical records, and patients who didn’t have a chart-documented diabetes diagnosis at enrollment may have been classified erroneously as not having diabetes. This could lead to potential bias in estimating the death risk between patients with and without diabetes. Hence, analysis of studies defining diabetes using rigorous guideline definitions of glucose measurement is needed to assess whether undiagnosed diabetes or newly diagnosed diabetes has any effect on post-AMI all-cause mortality.

### Conclusions

Our study presents evidence that diabetes has substantial and sustained effects on short-term, mid-term and long-term post-AMI all-cause mortality in both men and women. However, the risk of all-cause mortality associated with diabetes increased with longer follow-up in men; in women, the detrimental short-term, mid-term and long-term effects of diabetes on post-AMI all-cause mortality were similar. Although the summary adjusted risk ratio of all-cause mortality associated with diabetes appeared to be greater for women than for men at both short- and long-follow-up, this finding could be confounded by baseline differences in CVD risk factors, AMI-related characteristics, and other clinical profile. Considering the limitation of data gathered from the included studies, these findings should be regarded as hypothesis generating and stimulate future studies with robust strategies to elucidate the hypothesis. Nonetheless, the results highlight the need to tailor in-hospital and post-discharge care plans for patients with AMI based on their diabetes status, discharge duration, sex, and age. Furthermore, identifying individuals with undiagnosed diabetes and preventing the development of diabetes promises to have substantial effects on the prognosis of AMI.

## Data availability statement

The original contributions presented in the study are included in the article/**Supplementary Material** . Further inquiries can be directed to the corresponding author.

## Author contributions

QD, RW, ES, HL, JB, and MF contributed to the study design and editing the manuscript. JB contributed to the search strategy design and the literature search. QD performed the analyses and wrote the first draft of the manuscript. EW contributed to the analyses and interpretation. QD and MF are the guarantors of this work. All authors contributed to the article and approved the submitted version.

## Acknowledgments

The authors thank Purdue University librarian Jane Yacilla for her contribution to review the search strategy and re-run the updated literature search in PubMed.

## Conflict of interest

The authors declare that the research was conducted in the absence of any commercial or financial relationships that could be construed as a potential conflict of interest.

## Publisher’s note

All claims expressed in this article are solely those of the authors and do not necessarily represent those of their affiliated organizations, or those of the publisher, the editors and the reviewers. Any product that may be evaluated in this article, or claim that may be made by its manufacturer, is not guaranteed or endorsed by the publisher.
