# Supplementary material for: Sex-specific impact of diabetes on all-cause mortality among adults with acute myocardial infarction: An updated systematic review and meta-analysis, 1988-2021
Source: Front Endocrinol (Lausanne). 2022 Aug 17;13:918095. doi: 10.3389/fendo.2022.918095 (PMC9428712; doi:10.3389/fendo.2022.918095)
Supplement: Supplementary file 1 [file DataSheet_1.docx]

Supplementary Material

# Supplementary Data

Detailed search strategy:

Our final combined Medline and EmBase Search strategy are listed as follows:

| **#** | **Query** |
| --- | --- |
| 1 | exp Diabetes Mellitus/ |
| 2 | diabetes.ti,ab. |
| 3 | exp Myocardial Infarction/ |
| 4 | exp Acute Coronary Syndrome/ |
| 5 | heart attack.ti,ab. |
| 6 | AMI.ti,ab. |
| 7 | acute myocardial infarction*.ti,ab. |
| 8 | acute coronary syndrome.ti,ab. |
| 9 | exp Mortality/ or exp Survival Rate/ or exp Hospital Mortality/ or exp Fatal Outcome/ or exp "Cause of Death"/ |
| 10 | exp Death/ |
| 11 | exp Survival/ |
| 12 | outcome.ti,ab. |
| 13 | exp Mental Health/ |
| 14 | exp "Quality of Life"/ |
| 15 | exp Morbidity/ |
| 16 | exp Prognosis/ |
| 17 | health status.ti,ab. |
| 18 | functional status.ti,ab. |
| 19 | death.ti,ab. |
| 20 | mortality.ti,ab. |
| 21 | fatal.ti,ab. |
| 22 | survival.ti,ab. |
| 23 | 9 or 10 or 11 or 12 or 13 or 14 or 15 or 16 or 17 or 18 or 19 or 20 or 21 or 22 |
| 24 | 3 or 4 or 5 or 6 or 7 or 8 |
| 25 | 1 or 2 |
| 26 | 23 and 24 and 25 |
| 27 | sex.ti,ab. |
| 28 | gender.ti,ab. |
| 29 | (female or male).ti,ab. |
| 30 | exp Sex Factors/ |
| 31 | exp Sex Distribution/ |
| 32 | Female/ |
| 33 | Male/ |
| 34 | (women adj2 men).mp. |
| 35 | 27 or 28 or 29 or 30 or 31 or 32 or 33 or 34 |
| 36 | 26 and 35 |
| 37 | limit 36 to (English language and "all adult (19 plus years)" and (case reports or clinical study or clinical trial, all or clinical trial or comparative study or controlled clinical trial or meta analysis or observational study or pragmatic clinical trial or randomized controlled trial or "systematic review")) |

OVID EMbase Search Strategy

| 1 | exp diabetes mellitus/ |
| --- | --- |
| 2 | diabetes.ti,ab. |
| 3 | exp heart infarction/ |
| 4 | exp acute coronary syndrome/ |
| 5 | heart attack.ti,ab. |
| 6 | AMI.ti,ab. |
| 7 | acute myocardial infarction.ti,ab. |
| 8 | acute coronary syndrome.ti,ab. |
| 9 | exp mortality/ or exp "cause of death"/ or exp fatality/ or exp hospital mortality/ or exp survival rate/ |
| 10 | exp death/ |
| 11 | exp survival/ |
| 12 | outcome.ti,ab. |
| 13 | exp mental health/ |
| 14 | exp "quality of life"/ |
| 15 | exp morbidity/ |
| 16 | exp prognosis/ |
| 17 | health status.ti,ab. |
| 18 | functional status.ti,ab. |
| 19 | death.ti,ab. |
| 20 | mortality.ti,ab. |
| 21 | fatal.ti,ab. |
| 22 | survival.ti,ab. |
| 23 | 9 or 10 or 11 or 12 or 13 or 14 or 15 or 16 or 17 or 18 or 19 or 20 or 21 or 22 |
| 24 | 3 or 4 or 5 or 6 or 7 or 8 |
| 25 | 1 or 2 |
| 26 | 23 and 24 and 25 |
| 27 | sex.ti,ab. |
| 28 | gender.ti,ab. |
| 29 | (female or male).ti,ab. |
| 30 | exp sex factor/ |
| 31 | exp sex ratio/ |
| 32 | exp female/ |
| 33 | exp male/ |
| 34 | (women adj2 men).mp. |
| 35 | 27 or 28 or 29 or 30 or 31 or 32 or 33 or 34 |
| 36 | 26 and 35 |
| 37 | 13 or 14 or 17 or 18 |
| 38 | 24 and 25 and 35 and 37 |
| 39 | limit 36 to (English language and embase and (controlled clinical trial or multicenter study or phase 1 clinical trial or phase 2 clinical trial or phase 3 clinical trial or phase 4 clinical trial) and (adult <18 to 64 years> or aged <65+ years>)) |
| 40 | limit 38 to "reviews (best balance of sensitivity and specificity)" |
| 41 | 23 and 25 and 35 |
| 42 | limit 41 to (embase and "reviews (maximizes specificity)") |
| 43 | 24 and 42 |

# Supplementary Figures and Tables

## Supplementary Figures

**Supplementary Figure 1.** Funnel plot for short-, mid- and long-term all-cause mortality for women, using raw data from cohort studies

**
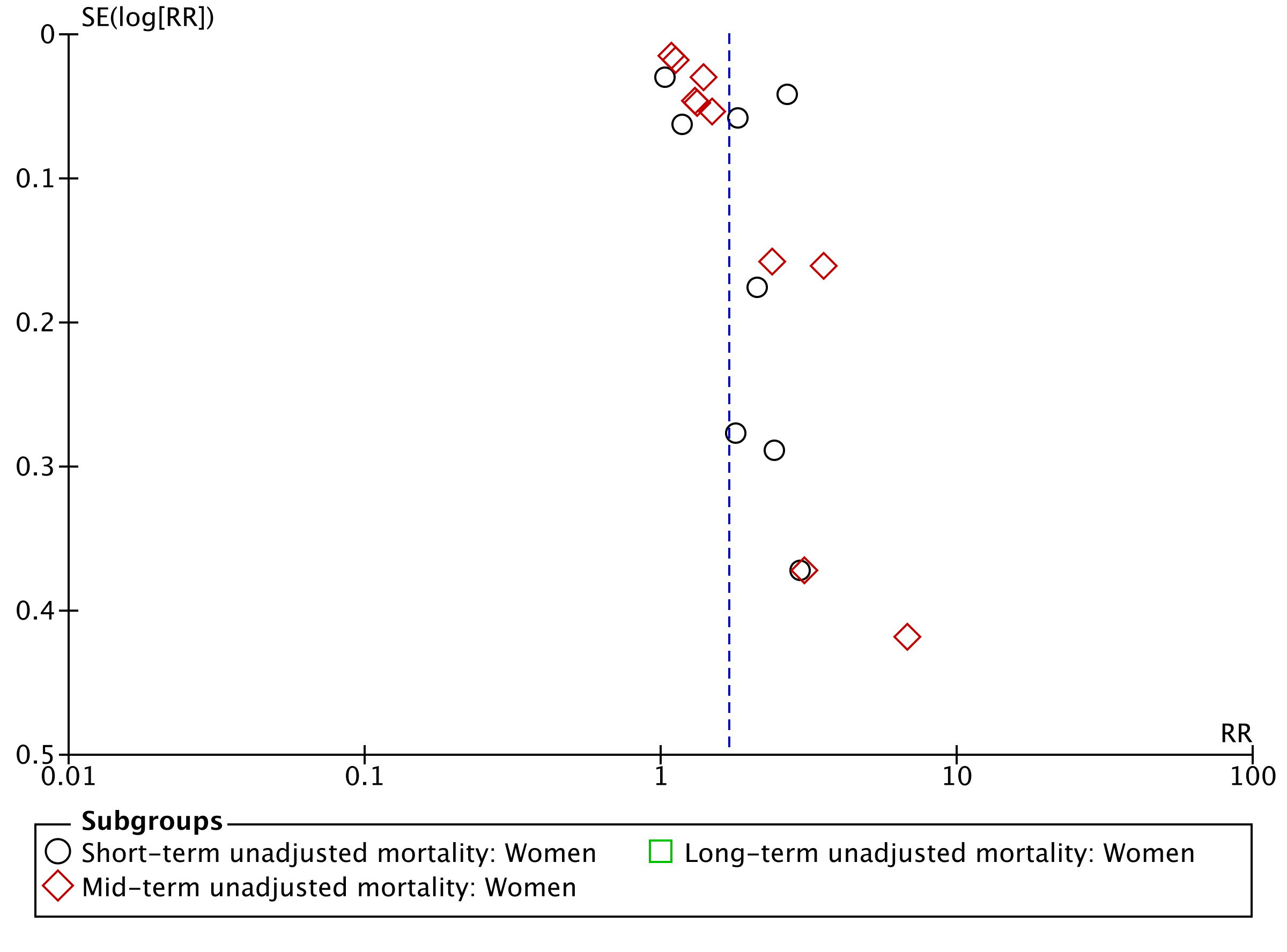
**

**Supplementary Figure 2.** Funnel plot for short-, mid- and long-term all-cause mortality for women, using adjusted data from cohort studies

**
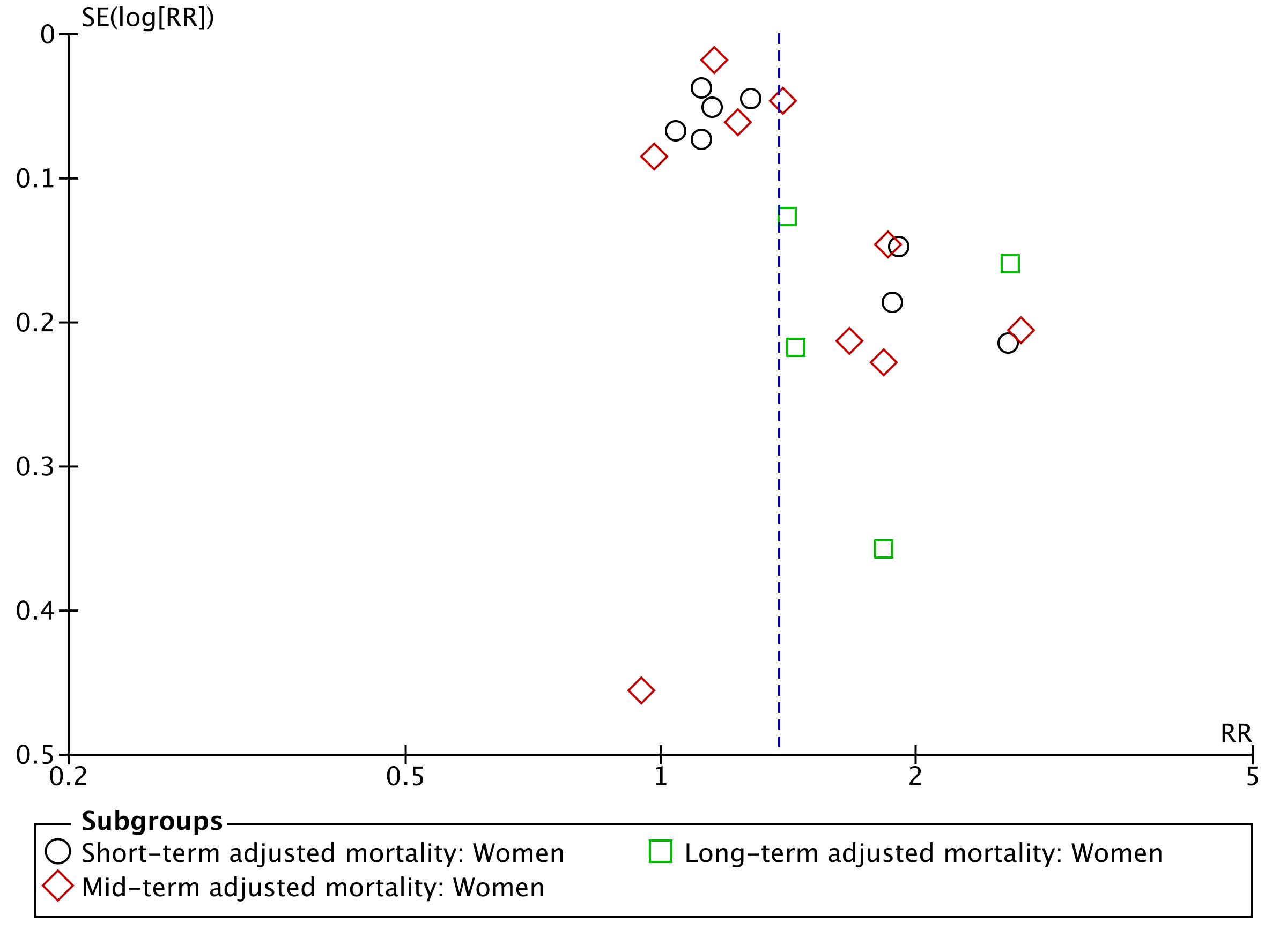
**

**Supplementary Figure 3.** Funnel plot for short-, mid- and long-term all-cause mortality for men, using raw data from cohort studies

**
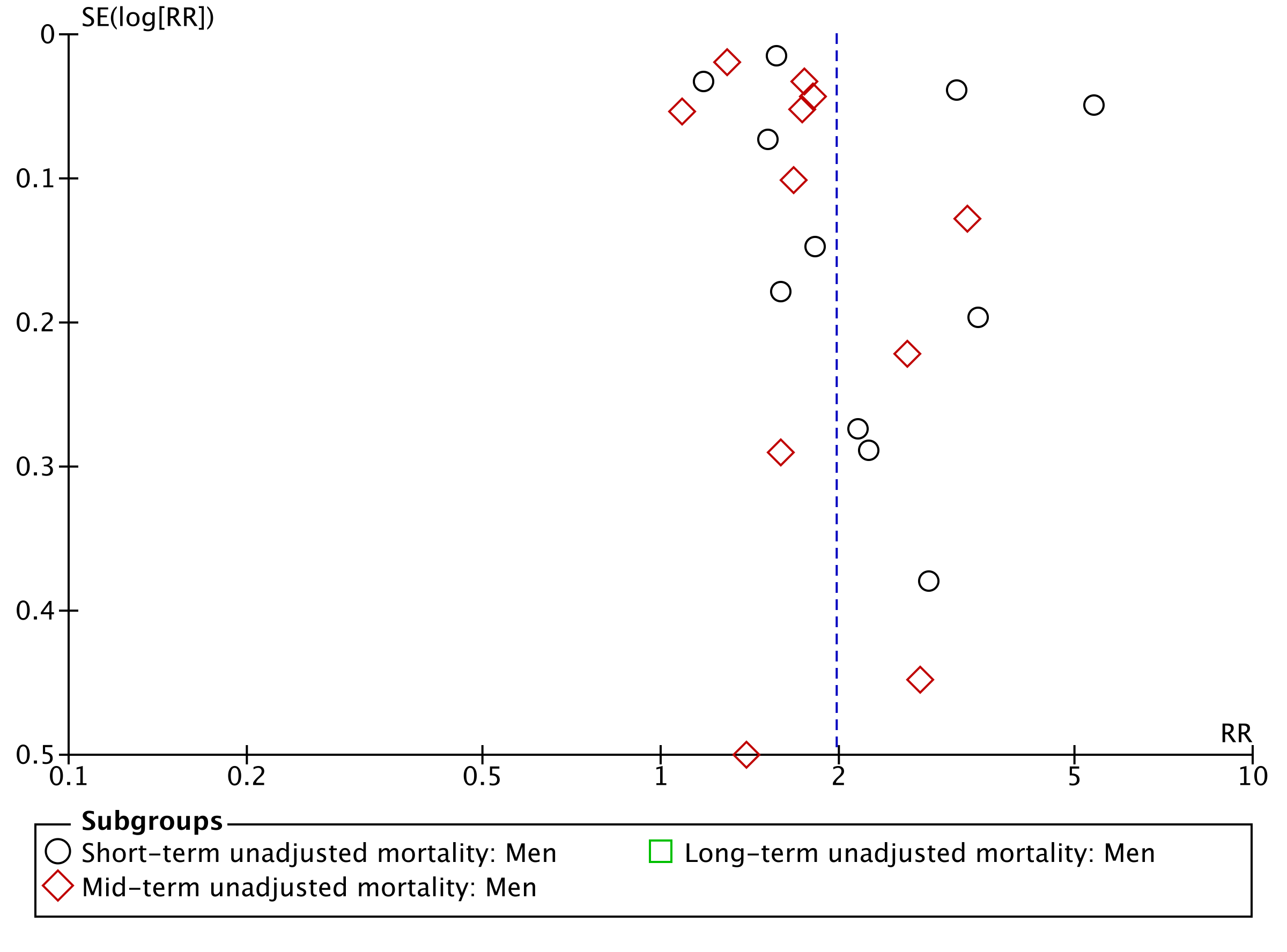
**

**Supplementary Figure 4.** Funnel plot for short-, mid- and long-term all-cause mortality for men, using adjusted data from cohort studies

**
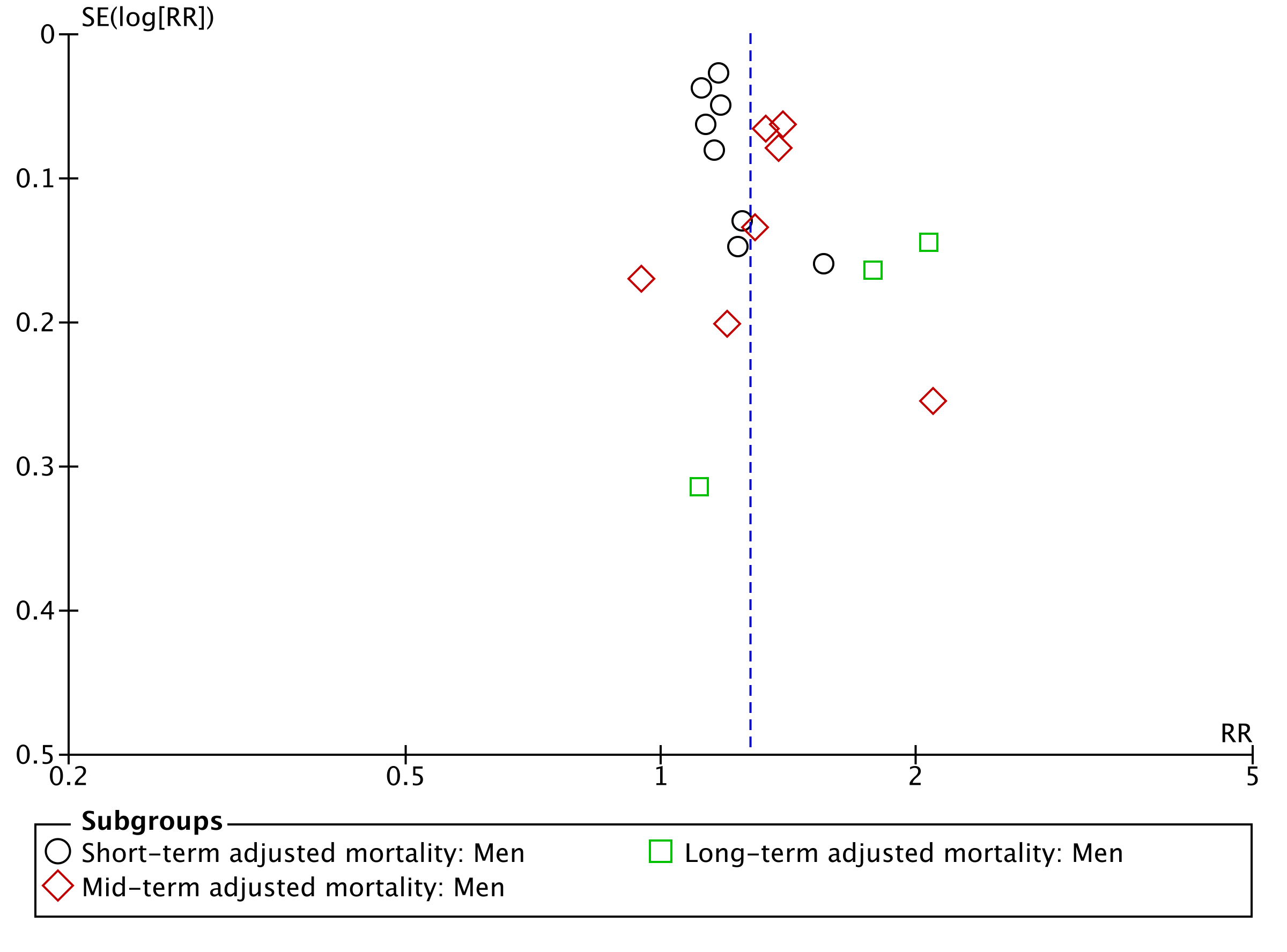
**

**Supplementary Figure 5.** Forest plots showing unadjusted short-, mid- and long-term post-AMI all-cause mortality when men with diabetes were compared with men without diabetes, using 19 cohort studies plus one randomized control study. IV indicates inverse variance; M-H, Mantel-Haensze; and RR, relative risk.


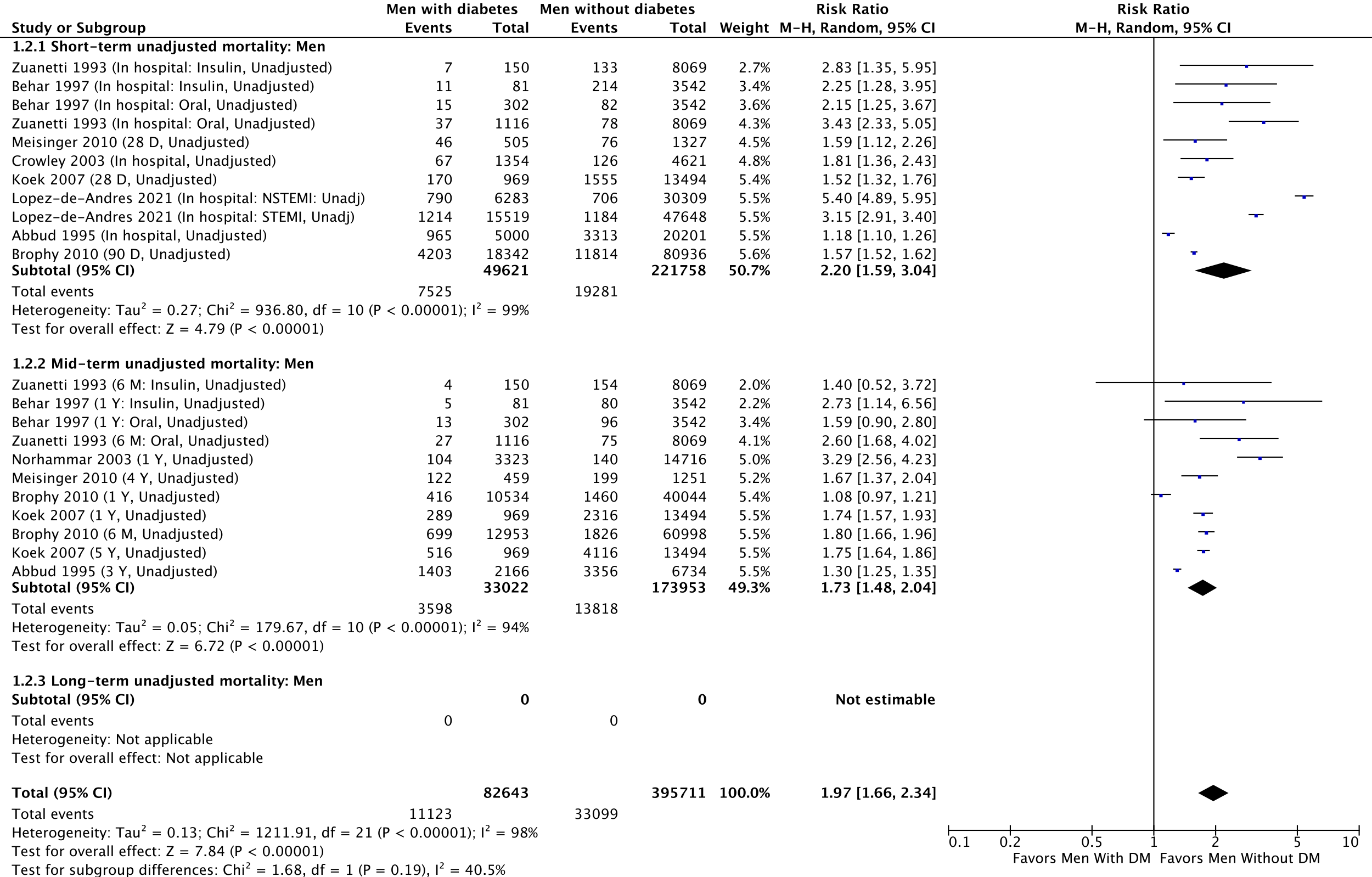


**Supplementary Figure 6**. Forest plots showing unadjusted short-, mid- and long-term all-cause mortality from 19 cohort studies plus one randomized control study when women with diabetes were compared with without. IV indicates inverse variance; M-H, Mantel-Haenszel; and RR, relative risk.

**
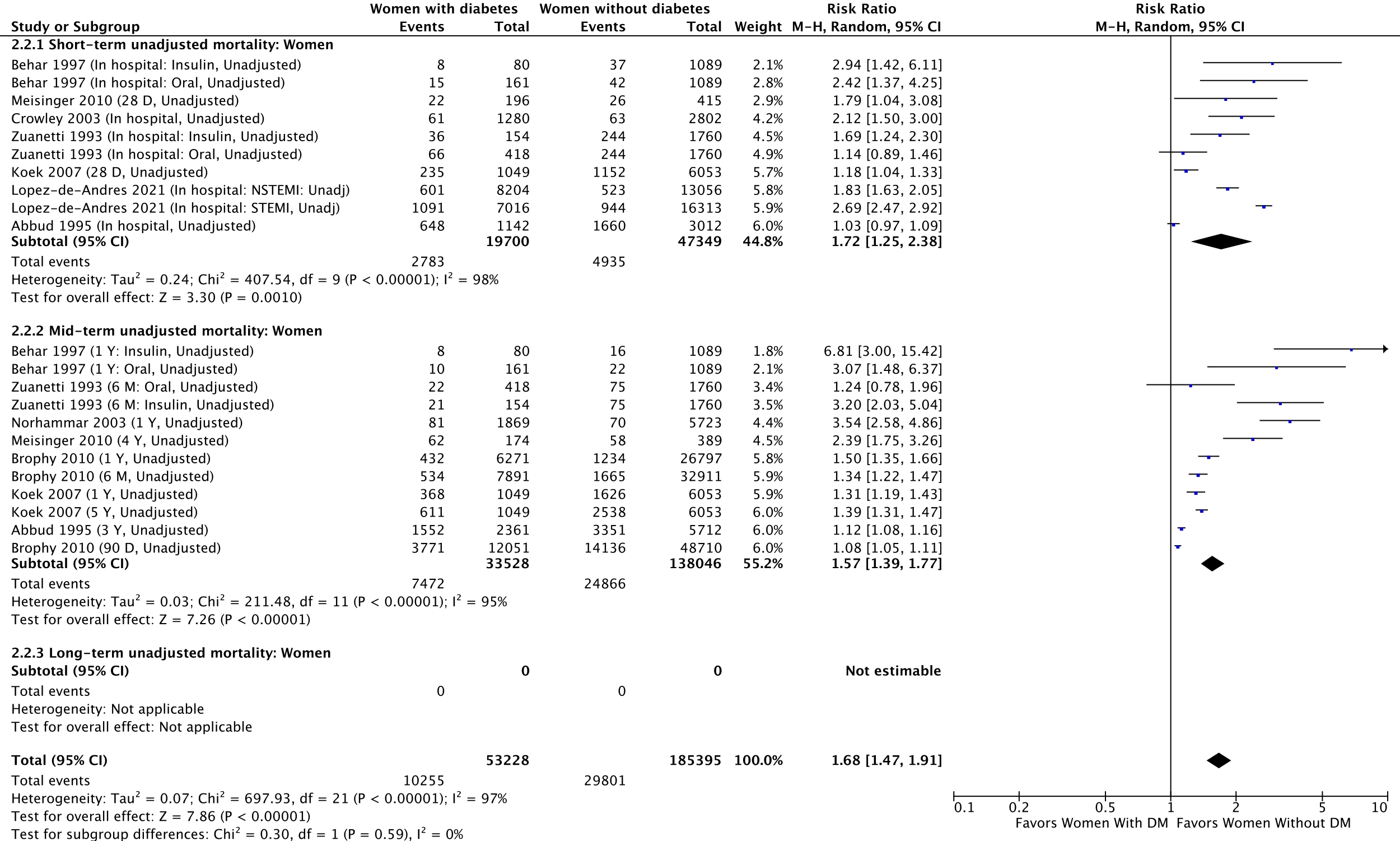
**

**Supplementary Figure 7.** Forest plots showing adjusted short-, mid- and long-term post-AMI all-cause mortality when women with diabetes were compared with women without diabetes, excluding studies highly influencing the pooled risk estimates

**
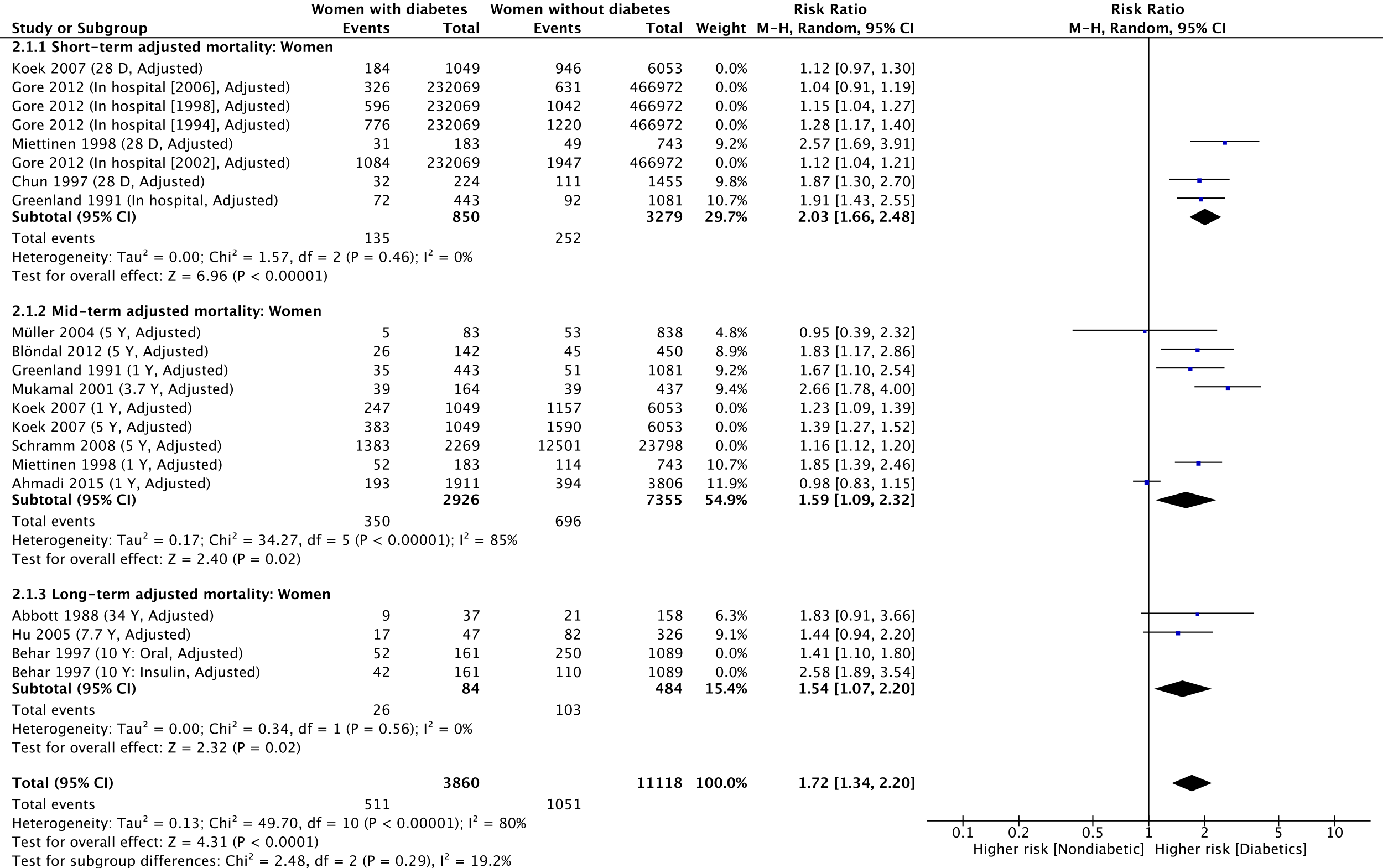
**

**Supplementary Figure 8.** Forest plots showing adjusted short-, mid- and long-term post-AMI all-cause mortality when men with diabetes were compared with men without diabetes, excluding studies influencing the pooled risk estimates.

**
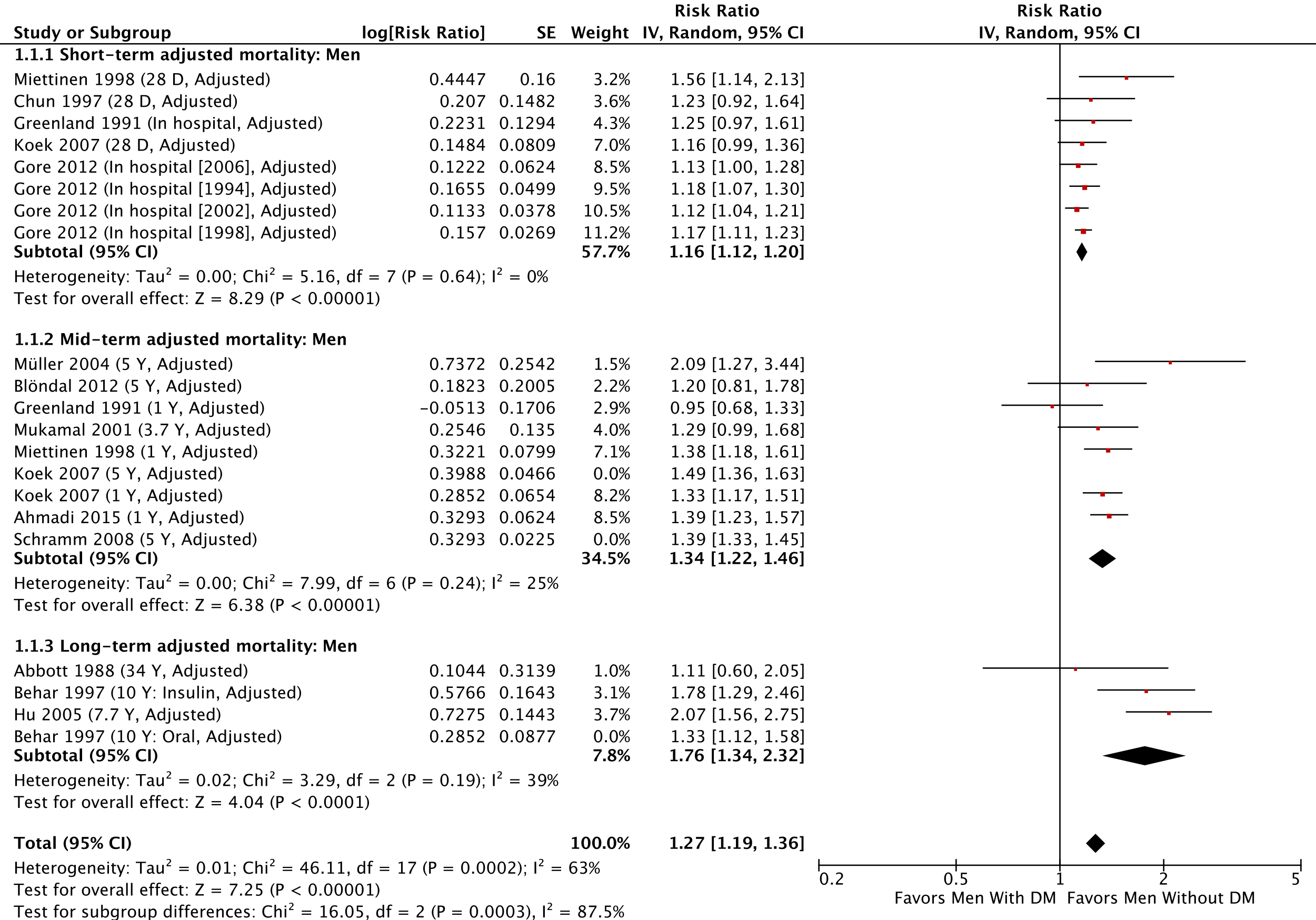
**

**Supplementary Figure 9.** Forest plot showing adjusted short-, mid- and long-term all-cause mortality when women with diabetes were compared with women with diabetes, stratified by mid-point of study period (before 2008 and from 2008). IV indicates inverse variance; and RR, risk ratio.

9.a. Study mid-point before 2008


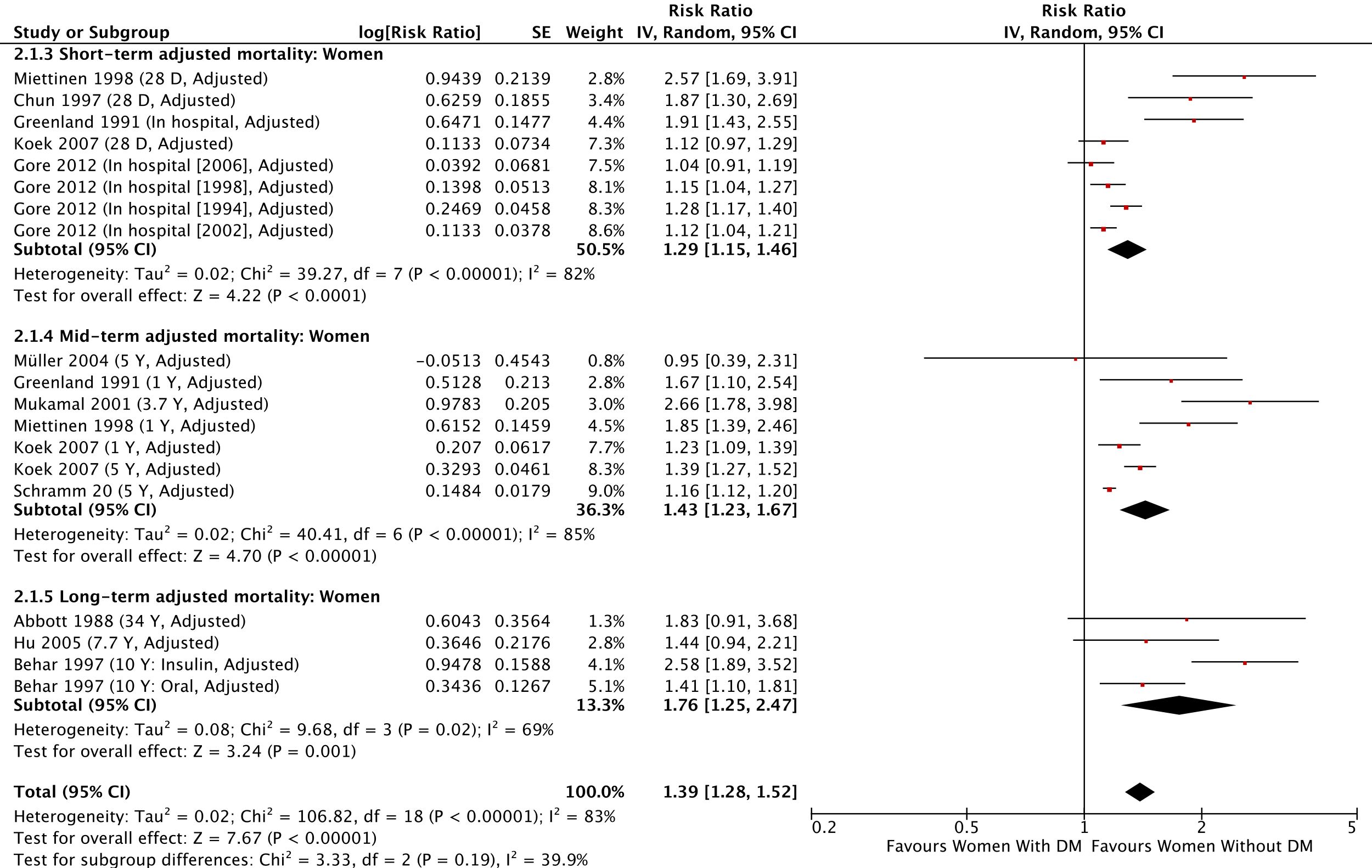


9.b. Study mid-point from 2008


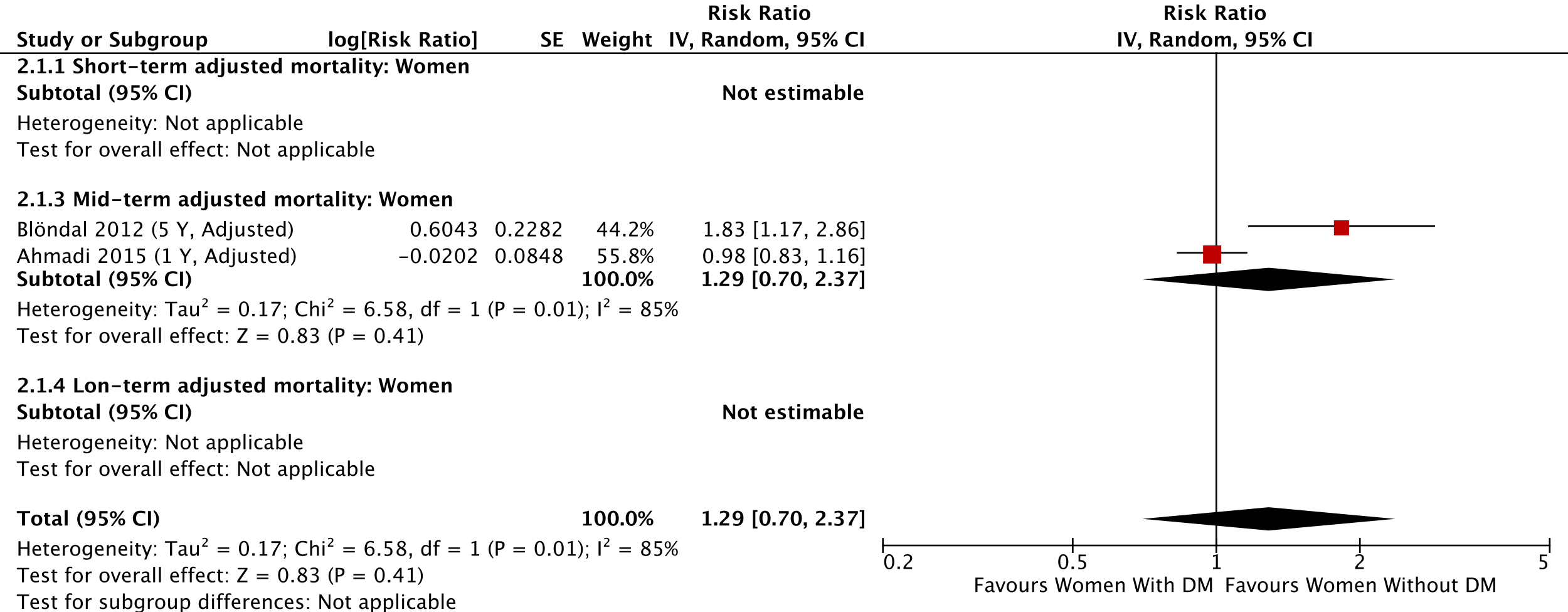


**Supplementary Figure 10.** Forest plot showing adjusted short-, mid- and long-term all-cause mortality when men with diabetes were compared with men with diabetes, stratified by mid-point of study period (before 2008 and from 2008). IV indicates inverse variance; and RR, risk ratio.

10.a. Study mid-point before 2008


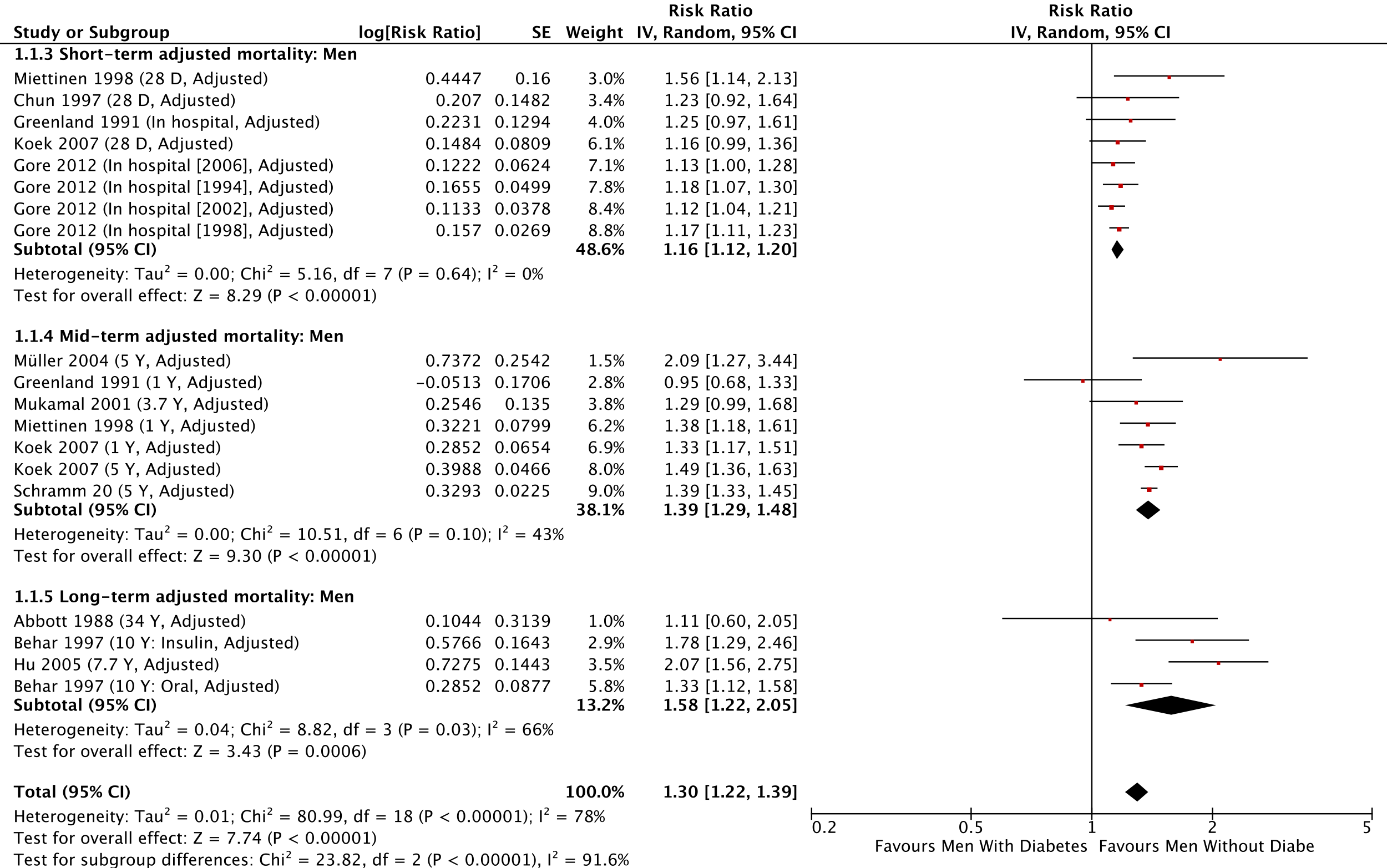


10.b. Study mid-point from 2008


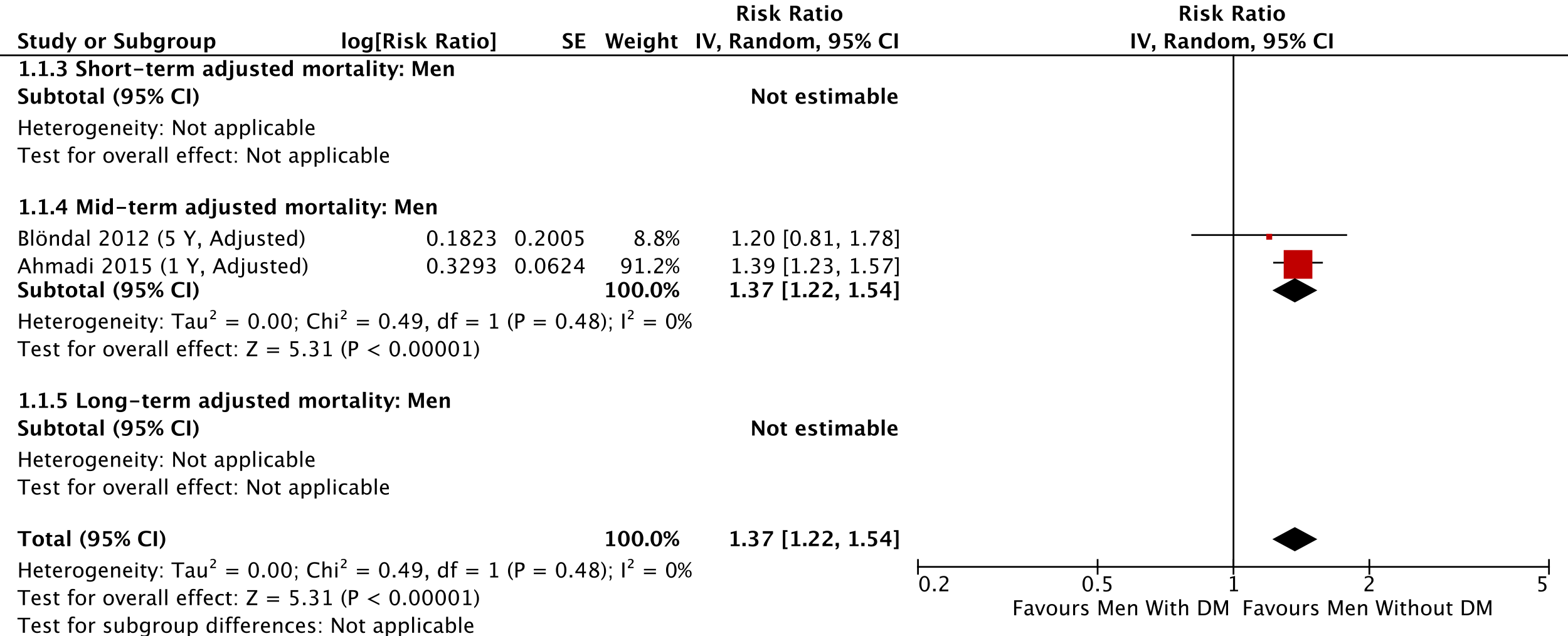


**Supplementary Figure 11.** Forest plot showing adjusted short-, mid- and long-term all-cause mortality when women with diabetes were compared with women with diabetes, stratified by mean age (<65 years and 65 years or older). IV indicates inverse variance; and RR, risk ratio.


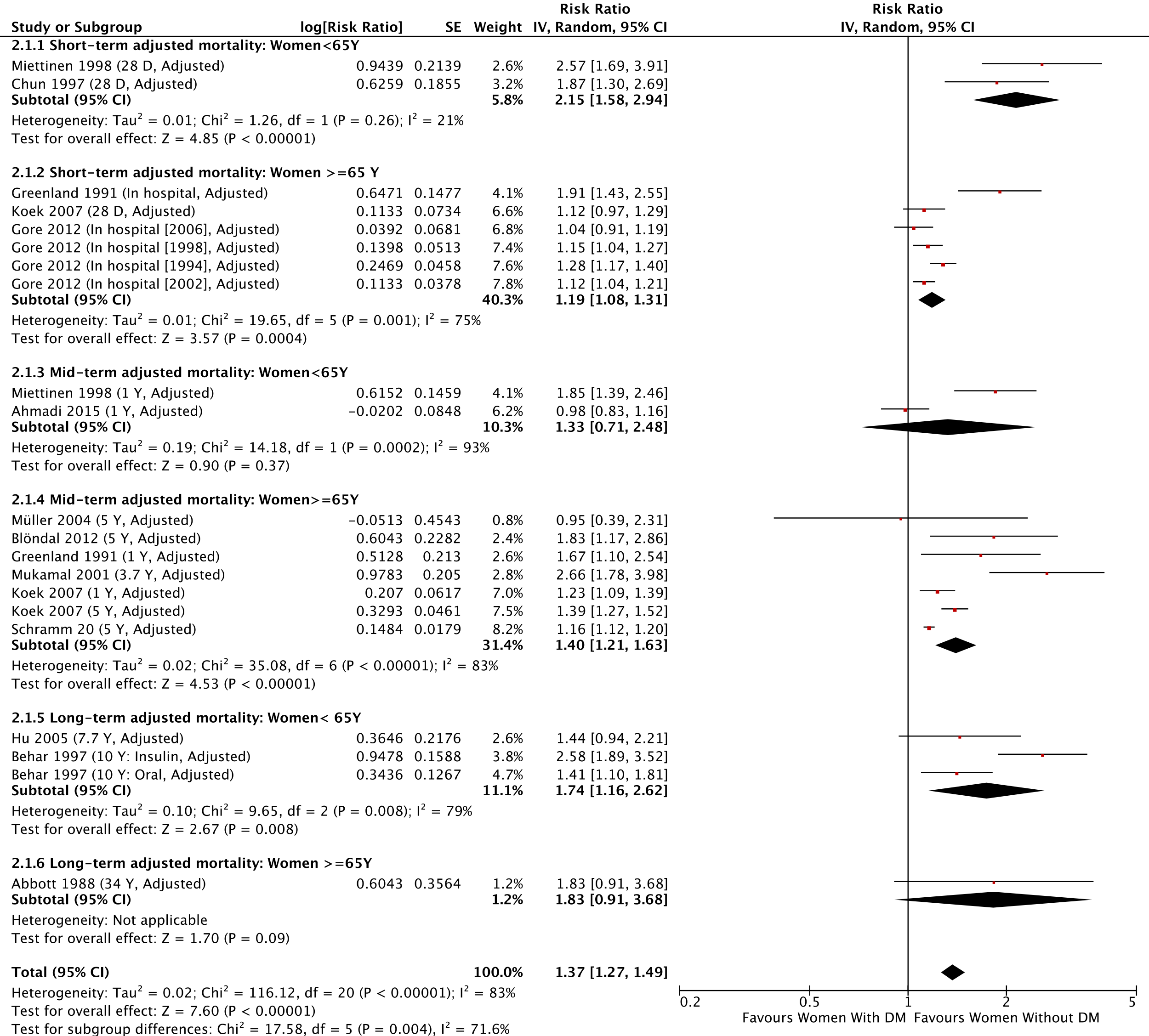


**Supplementary Figure 12.** Forest plot showing adjusted short-, mid- and long-term all-cause mortality when men with diabetes were compared with men with diabetes, stratified by mean age (<65 years and 65 years or older). IV indicates inverse variance; and RR, risk ratio.


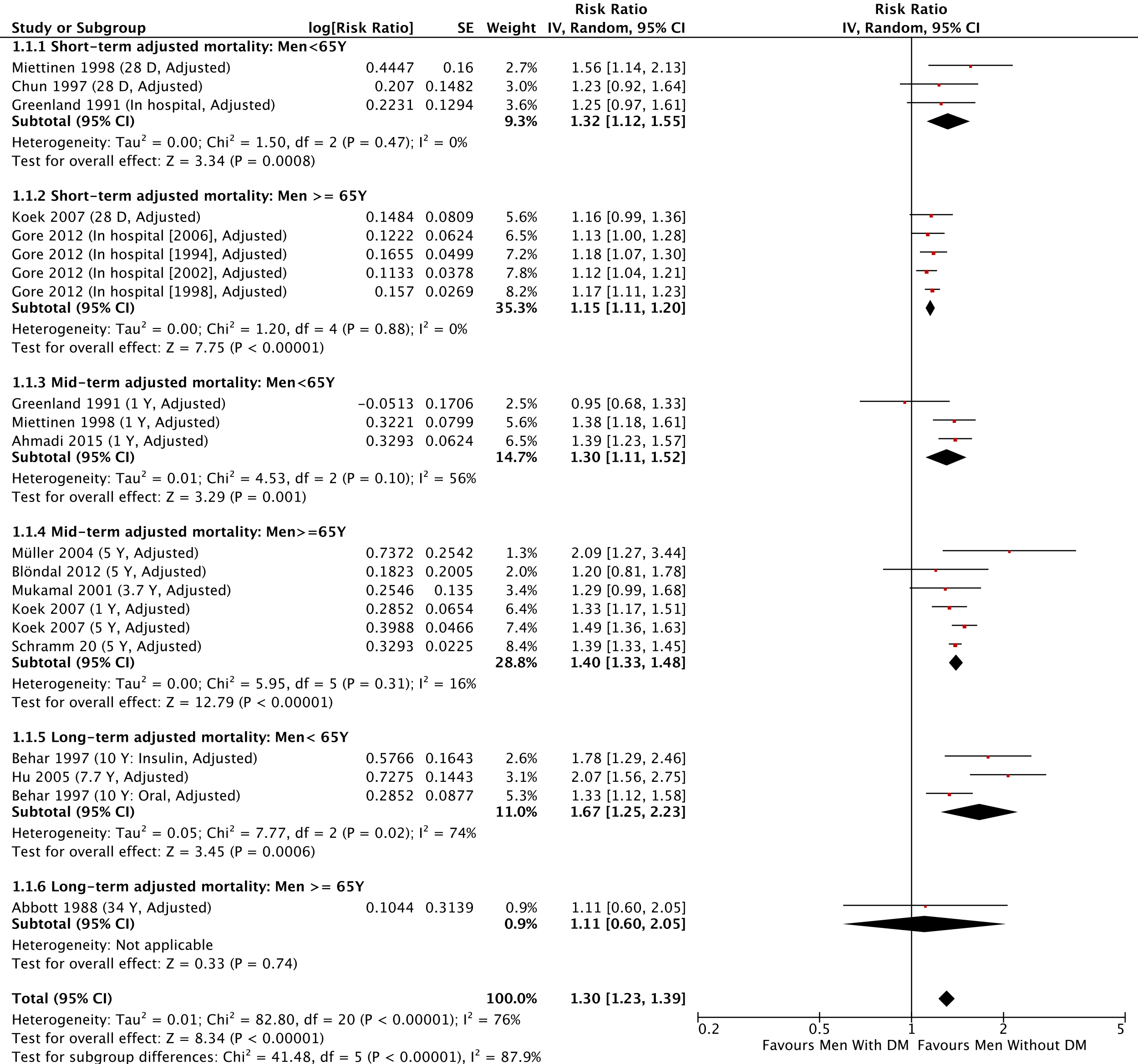


## Supplementary Tables

**Supplementary Table 1.** Subgroup and Sensitivity Analyses

| Sex | Adjustment Type | Analysis restricted to | Relative risk of association (With diabetes vs. Without diabetes) | | |
| --- | --- | --- | --- | --- | --- |
|  |  |  | Short-term mortality | Mid-term mortality | Long-term mortality |
| Sensitivity analysis | | | | | |
| Women | Unadjusted | Data from cohort studies | 1.83 [1.26-1.2.67] | 1.52 [1.34-1.72] | NA |
| Women | Unadjusted | Data from cohort studies +1RCT* | 1.72 [1.25-2.38] | 1.57 [1.39-1.77] | NA |
| Men | Unadjusted | Data from cohort studies | 2.06 [1.45-2.93] | 1.69 [1.43-2.00] | NA |
| Men | Unadjusted | Data from cohort studies +1RCT* | 2.20 [1.59-3.04] | 1.73 [1.48-2.04] | NA |
| Subgroup analysis | | | | | |
| By age | | | | | |
| Women | Adjusted | <65 years | 2.15 [1.58-2.94] | 1.33 [0.71-2.48] | 1.74 [1.16-2.62] |
| Women | Adjusted | >=65 years | 1.19 [1.08-1.31] | 1.40 [1.21-1.63] | 1.83 [0.91-3.68]^ |
| Men | Adjusted | <65 years | 1.32 [1.12-1.55] | 1.30 [1.11-1.52] | 1.67 [1.25-2.23] |
| Men | Adjusted | >=65 years | 1.15 [1.11-1.52] | 1.40 [1.33-1.48] | 1.11 [0.60-2.05]^ |
| By mid-point of study period | | | | | |
| Women | Adjusted | Before 2008 | 1.29 [1.15-1.46] | 1.43 [1.23-1.67] | 1.76 [1.25-2.47] |
| Women | Adjusted | From 2008 | NA | 1.29 [0.70-2.37] | NA |
| Men | Adjusted | Before 2008 | 1.16 [1.12-1.20] | 1.39 [1.22-2.05] | 1.58 [1.22-2.05] |
| Men | Adjusted | From 2008 | NA | 1.37 [1.22-1.54] | NA |

Abbreviations: NA=information not available in the published study paper or supplemental material; RCT=randomized control trial; NA=data not available

Note: *The 1 RCT trial included only provided unadjusted data

^Only one study reported sex-specific long-term (>5 years) follow-up mortality data

Supplementary Table 2. Clinical and methodological characteristics of included studies.

| **Study** | **Study participants characteristics** | **Hospital characteristics** | **Baseline myocardial infarction severity** | **Baseline diabetes related characteristics** | **AMI interventions/ procedures received** | **Study design** | **Study conduct** | **Statistical approach differences** |
| --- | --- | --- | --- | --- | --- | --- | --- | --- |
| **Abbott et al., 1988[1]** | AMI survivors in Framingham Study | NR | NA | - Type 1 or 2 diabetes unspecified - Diabetes defined by history, use of antidiabetic medications, or lab values confirmed by glucose tolerance tests | NA | Longitudinal cohort study | Retrospective analysis of observational study | - Estimates of RRs were based on corresponding regression coefficient - RR adjusted risk factors measured at most recent examination after initial AMI |
| **Abbud et al., 1995[2]** | Patients with myocardial infarction discharged from 90 nonfederal hospitals in New Jersey | Nonfederal hospitals in New Jersey | NA | - The presence or absence of type 2 diabetes - (ICD-9 250.00 to 250.90) | NA | Observational cohort study | Statewide database extraction | Proportional hazards regression excluding patients with kidney disease & cancer |
| **Ahmadi et al., 2015[3]** | Patients hospitalized with a new presentation with MI (across 540 hospitals) in Iran | 540 hospitals in Iran | - 75.8% STEMI-ST-(segment elevation MI) - Anterior MI (20.9%) - Inferior MI (34.6%) | With and without type 2 diabetes | - Coronary artery bypass grafting (CABG) (2.6%) - Percutaneous coronary intervention (PCI) (6.9%) - Lack of thrombolytic therapy (44.5%) | Retrospective cohort study | Prospective analysis of data from the MI Registry of Iran’s Cardiovascular Diseases Surveillance System | Chi square, t test, Cox and logistic regression |
| **Behar et al., 1997[4]** | AMI patients hospitalized in 13 coronary care units in Israel | Coronary care unit in Israel | History of MI (26% in men; 19% in women) | History of diabetes or physician diagnosis of insulin and non-insulin dependent diabetes | NA | Registry cohort | Data extraction obtained from patients who were included in a secondary prevention trail using SPRINT | Logistic regression and Cox proportional hazard model |
| **Blondal et al., 2012[5]** | Patients hospitalized with diagnosis of AMI in the Estonian MI Registry (EMIR), the Estonian Health Insurance Fund (EHIF) database, and Estonian Population Registry (EPR) | Tertiary care PCI center | 3-4 vessel disease (43.7% in non-DM in men, 54.2% in DM for men; 45.3% in Non-DM in women, 64.1% in DM for women) | - History of diabetes or physician diagnosis - Unknown type of diabetes | All included patients received percutaneous coronary interventions (PCI) | Register linkage study | Data analysis of linked registers in Estonia | Procedural characteristics of PCI, prescription of medication, and outcomes the Chi-square test for categorical variables and t-test for two independent samples or the Wilcoxon-Mann-Whitney test for continuous variables were used to compare diabetic vs non-diabetic patients by sex in respect to baseline characteristics |
| **Brophy et al., 2010[6]** | MI patients hospitalized with an MI in England and Wales (patient less than 30 years were excluded) | NA | NA | ICD codes E10-E14 (insulin dependent, non-insulin dependent, malnutrition-related DM, other specified diabetes, and other non-specified diabetes) | Proportion had any heart surgery (Angioplasty, CABG, or stent placement): 15.3% in diabetes; 16.2% in non-diabetes | Cohort study | Data analysis of linked registers | - STATA used to examine survival for patients stratified by characteristics (age, sex, diabetes) - Factors (diabetes, heart failure, age, gender, and surgery for the current AMI) were analyzed using Cox regression - Proportionality was examined by comparing graphs of the scaled Schoenfeld residuals |
| **Chun et al., 1997[7]** | Patients with first AMI (aged between 30-69) in Newcastle, New South Wales, Australia | NA | NA | - Insulin and/or oral hypoglycemic agents before the acute event - Patients not previously known to be diabetic who were diagnosed during hospital admission and discharged with persisting diabetes - No information about the type of diabetes | NA | Cohort study | Data extraction based on a community-based surveillance system that monitors coronary heart disease morbidity and morality | Age-adjusted case fatality, prevalence of diabetes, and other risk factors calculated by direct method using age distribution of all study patients as the standard population. Univariate comparison between patients with and without diabetes assessed using the chi-squared test for categorical variables; Wilcoxon’s test used to compare medians due to skewed distribution of patient ages; multiple logistic regression used to assess combined effects of diabetes and other risk factors on case fatality |
| **Crowley et al., 2003[8]** | Community-wide study of residents of Worcester, Mass. who were hospitalized with AMI | All 16 hospitals in Worcester | Patients with peri-operative AMI were excluded; Anterior AMI (47.9% in men, 52.9% in women) | - History of diabetes in medical records - Unknown diabetes types | - Cardiac catheterization (26.7% in men, 18.7% in women) - CABG (4.2% in men, 3.1% in women) - Percutaneous transluminal coronary angioplasty (PTCA) (9.7% in men, 6.2% in women) | Multi-hospital observational study | Data abstraction through individual reviews and validations of medical records according to pre-defined diagnostic criteria | Multiple logistic regression analysis used to assess independent effect of sex on hospital case-fatality rates and 1-year post-hospital-discharge death rates. Kaplan Meier survival plots for differences in long-term survival rates between men and women with DM were compared using log rank test |
| **Gore et al., 2012[9]** | MI patients treated in 1946 hospitals in the U. S | Acute hospitals | ST-segment elevation MI or left bundle-branch block: 45.6% in all | History of diabetes in medical records or patient self-report | Percutaneous coronary intervention (8.0%), coronary artery by-pass grafting (13.5%); reperfusion therapy in diabetic vs. non-diabetic: 59% vs. 72% | Observational study | Data analysis represents participant from approximately 1/4 of US acute care hospitals | Summary data and inferential statistics were weighted based on a 12-category cross-classification of hospital facility type and number of beds to correct for changes in characteristics of participating centers over time |
| **Greenland et al., 1991[10]** | Patients with acute MI in 14 hospitals throughout Israel | Coronary care units of participating hospitals | - Prior MI (18.5% in women, 26.9% in men) - Angina before MI (50.7% in women, 48.7% in men) - Anterior MI (44% in women, 44.1% in men) - Elevated cardiac enzymes (57.6% in women, 65.7% in men) | Medical records without known type of diabetes | NA | Cohort study | Demographic and medical data of patients were collected during hospitalization and 1 year after discharge | - Mantel-Haenszel test used to compare age-adjusted death rates - Logistic regression used to estimate covariate adjusted odds ratios associated with female gender - 95% CI approximated for ORs by taking the antilogs of the logistic coefficients ±1.96SE - Separate logistic regression analyses performed for women, men, nondiabetics |
| **Hu et al., 2005[11]** | Patients with prior diabetes or MI (aged 25-64 years) in 5 geographic areas of Finland | NA | Hospital discharge diagnosis of MI before baseline survey | - Self-report or medical record and confirmed by physician - Unknown diabetes types | NA | Cohort studies | Six independent population surveys were carried out in 5 geographic areas of Finland in 1972, 1977, 1982, 1987, 1992, 1997 | - Gender-specific CHD, CVD, non-CVD and total mortality rates calculated by 10-year age intervals and standardized for age by the direct method using a European standard population age 25-74 years for each of the 3 groups: diabetes, MI, and both - Cox proportional hazard models used to estimate the HRs of cause-specific and all-cause mortality associated with diabetes and MI status - Likelihood ratio test for interaction used to test if the effect of disease status on mortality was the same in men and women |
| **Koek et al., 2007[12]** | First AMI in Netherlands | All general and academic hospitals and most specialty hospitals in the Netherlands | Prior admission for CVD in patients with diabetes (men: 31.7%, women: 31%); in patients without diabetes (men 16.8%, women 16.3%) | Diabetes identified by ICD-9 codes | NA | Register linkage study | Data analysis of patients first hospitalized for AMI in 1995 in hospital register in the Netherlands | - For diabetic and nondiabetic patients, crude short-term, 1 year, and long-term mortality risks were computed - Cox proportional hazard analyses used to examine association between DM and overall survival in men and women - Cox proportional hazards models used to determine if gender, age, previous cardiovascular disease, and ethnic origin were independently associated with overall survival for patients with and without DM |
| **Lopez-de-Andres et al., 2021[13]** | STEMI and NSTEMI patients in Spain | All hospitals in Spain | STEMI vs NSTEMI | Type 2 diabetes per ICD 10 | - CABG (In men : with DM : 1.2%, no DM : 1.5% ; in women with DM : 0.6%, no DM : 0.7%). - PCI during hospitalization (in men without DM : 62.1%, men with DM : 60.7% ; in women without DM : 48.4%, women without DM : 46.6%). | Observational study | Data analyzed of all patients 40+ years of age in years 2016, 2017, and 2018 who were discharged with a primary diagnosis of MI (STEMI and NSTEMI). | Conducted separately for women and men:   - Poisson regression models used to analyze incidence adjusted for age and sex when required - t-test or Mann-Whitney test used to compare continuous variables - Chi-square test used to compare categorical variables - AORs obtained using multivariable logistic regression to identify variables independently associated with IHM - Effect of sex analyzed using entire database. |
| **Meisinger et al., 2010** [14] | First AMI in region of Augsburg | NA | Anterior wall infarction (men without DM: 49%, men with DM: 41%; women without DM: 48%, women with DM: 50%) | - Self-reported - Unknown diabetes type | - PCI (Men without DM: 53%, men with DM:43%; women without 47%, women with DM 34%). - CABG (men without DM: 16%, men with DM 18%; women without DM: 13%, women with DM: 16%). - Any reperfusion therapy (in men without DM: 80%, men with DM: 71%; women without DM: 72%, women with DM: 60%). | Population based study | AMI registry | - Univariate survival analysis was performed using Kaplan-Meier curves. - Comparisons between survival curves were performed using log-rank test. |
| **Miettinen et al., 1998[15]** | Finnish contribution to WHO MONICA project (25-64 with a suspected MI or died of CHD) | Local health center, regional hospital, central hospital | Anterior infarction (nondiabetic: 48%, diabetic: 47%) | - Diabetes diagnosed during AMI hospitalization - Unknown type of diabetes | Fibrinolytic therapy (21% in nondiabetic, 13% in diabetic) | Cohort study | - FINMONICA - Myocardial Infarction Register | - Mantel-Haenszel procedure for categorical variables - General linear models for continuous variables - Kaplan-Meier product-limit method and standard life-table methods used to calculate survival probability curves - Cox proportional-hazards regression model used to calculate HRs |
| **Mukamal et al., 2001[16]** | Patients hospitalized with a confirmed AMI at 45 U.S. medical centers | Community based and tertiary hospitals | - Q-wave infarction (52% in diabetic, 57% in non-diabetics) - Prior MI (39% in diabetic, 26% in non-diabetic) | Defined DM based on chart review or use of hypoglycemic medication, provided type of diabetes, duration of time from diagnosis of diabetes to onset of AMI | Thrombolytic therapy (28% in diabetics, 385 in non-DM) | Prospective cohort study | Eligible patients were identified through review of coronary care unit admission logs and patient charts | - Student’s t tests and exact tests used to analyze continuous and binary variables - Log-rank test used to compare unadjusted Kaplan-Meier survival plots - Cox proportional hazards models used to examine effect of diabetes on survival after adjustment for potential confounding factors |
| **Muller et al., 2004** [17] | Patients admitted with Unstable angina or non-Segment elevation MI | A heart center in Germany | - New ST depression at entry (diabetic 14%; non-diabetic 10%) - Prior MI (41% in diabetic, 31% in non-diabetic) - Three coronary vessels with more than 50% stenosis (53% in diabetic, 35% in non-diabetic) | - DM defined by known history or treatment for diabetes - Unknown type of diabetes | - PCI (53% in diabetic, 56% non-diabetic) - CABG (21% in diabetic, 12% in non-diabetic) | Prospective cohort study | Analysis was performed on consecutive patients admitted to center with UA/NSTEMI who were treated with a very early invasive surgery | - Wilson method used to calculate CI for rarer outcomes - Comparison made using ANOVA for independent samples and chi-square tests - Cox proportional hazards regression analysis used as appropriate method throughout study - Multivariate Cox regression analysis used to identify independent predictors of death - Kaplan-Meier method used to construct cumulative survival curves |
| **Norhammar et al., 2003** [18] | AMI patients below 80 years admitted to coronary care units at 58 hospitals in Sweden | 58 participant hospitals with coronary care units in Sweden | Prior MI (non-diabetic: 21%, diabetic 33%) | Diabetes mellitus was based on either information from the patient of the disease at hospital admission or the prescription of insulin or oral anti-diabetic drugs at admission and/or at discharge | - Reperfusion therapy (non-DM: 41%, DM: 31%) - Revascularization (non-DM: 5%, DM: 4%) | Cohort study | Analysis performed on patients registered through the Register of Information and Knowledge about Swedish Heart Intensive Care Admissions (i.e., patients admitted to coronary care units of all participating hospitals) | - Chi-square tests for categorical variables - Student’s t-test for continuous variables - Bivariate analyses and multiple covariate Cox regression analyses used to identify variables with significant influence on treatment and mortality |
| **Schramm et al., 2008[19]** | All inhabitants in Denmark 30 years and older, and compared between patients with diabetes requiring glucose lowering medication versus non-diabetic patients with and without a prior MI | NR | Prior MI (No diabetes: 2.3%, diabetics: 8.9%) | - Individuals claiming having at least 1 prescription of glucose lowering medication - Unknown type of diabetes | NA | Population cohort | Individual-level linkage of nationwide registers in Denmark used to identify residents over 30 years of age for 5 years of follow-up | - Cox multivariable proportional-hazard regression models used to estimate differences among risk groups - Cox analyses used with adjustment for age and with multivariable adjustment for age, gross income, comorbidity, and time-dependent adjustment for medical treatment during follow-up |
| **Zuanetti et al., 1993[20]** | Patients enrolled in GISSI-2 study; all patients received fibrinolytic agents | Tertiary | - Patients with previous MI (16% in men; 9.1% in women) - Anterior MI (31.4% in men, 34.4% in women) | Insulin-dependent and non-insulin dependent diabetes; both type 1 and type 2 included | Randomized to receive Streptokinase with or without heparin, or recombinant tissue-type plasminogen activator (rt-PA) | RCT | Analysis on patients enrolled in GISSI-2 study | - Chi-square statistic used to test statistical significance of differences for genders in prevalence of concomitant diseases according to patient diabetic status - Chi-square statistics used to compare in-hospital mortality and total and sudden mortality rates after 6 months of follow-up - Mantel-Haenszel OR used to express results |

Abbreviations:

NA: not available

NR: not relevant

AMI: acute myocardial infarction

RR: relative risk

CABG: coronary artery bypass grafting

PCI: percutaneous coronary intervention

MI: myocardial infarction

DM: diabetes mellitus

PTCA: percutaneous transluminal coronary angioplasty

CI: confidence interval

SE: standard error

CVD: cardiovascular disease

STEMI: ST-elevation myocardial infarction

NSTEMI: non-ST-elevation myocardial infarction

AOR: adjusted odds ratio

IHM: in-hospital mortality

rt-PA: recombinant tissue-type plasminogen activator

RCT: randomized controlled trial

OR: odds ratio

**Supplementary Table 3.** Sex-specific short-, mid- and long-term mortality associated with diabetes after myocardial infarction.

| **Adjustment types** | **Follow-up duration** | **Men (Adults with diabetes versus without diabetes)** | **Women (Adults with diabetes versus without diabetes)** | **Men (Adults with diabetes versus without diabetes)** | **Women (Adults with diabetes versus without diabetes)** | **Variables adjusted in analysis** | **Diabetes definition** |
| --- | --- | --- | --- | --- | --- | --- | --- |
|  |  | **Unadjusted RR (95% CI)** | **Unadjusted RR (95% CI)** | **Multivariate adjusted RR**  **(95% CI)** | **Multivariate adjusted RR**  **(95% CI)** |  |  |
| **Sex-specific short-term mortality** | | | | | | | |
| **Abbud et al., 1995** | In-hospital | 1.18 (1.10, 1.26) | 1.03 (0.97, 1.09) | NA | NA | None | Medical record |
| **Behar et al., 1997** | In-hospital: Insulin treatment | 2.25 (1.28, 3.95) | 2.94 (1.42, 6.11) | NA | NA | None | History of diabetes, physician diagnosis |
| **Behar, 1997** | In-hospital: Oral diabetic treatment | 2.15 (1.25, 3.67) | 2.42 (1.37, 4.25) | NA | NA | None |  |
| **Chun, 1997** | 28 days post discharge | NA | NA | 1.23 (0.92, 1.65) | 1.87 (1.30, 2.70) | - Demographics (Age)  - CVD risk factors (Smoking, hypertension, high cholesterol) | History of diabetes documented by a physician; patients diagnosed with diabetes during the current episode |
| **Crowley et al., 2003** | In-hospital | 1.81 (1.36, 2.43) | 2.12 (1.50, 3.00) | NA | NA | None | Based on review of data contained in medical records with no independent confirmation of diabetes diagnosis |
| **Gore et al., 2012** | In-hospital [1994 data] | NA | NA | 1.18 (1.07, 1.30) | 1.28 (1.17, 1.40) | -Demographics (Age, race)  - CVD risk factors (Smoking status, history of hypertension, family history of coronary artery disease, history of chronic heart failure, medical history of stroke, medical history of angina)  - AMI-related characteristics (previous MI, Killip class, ST-segment elevation or left bundle-branch block, anterior MI, chest pain at presentation, prior percutaneous coronary intervention, prior coronary artery by-pass grafting,)  - Other clinical characteristics (systolic blood pressure, and pulse rate) | Medical record or patient information at the time of hospitalization |
| **Gore et al., 2012** | In-hospital [1998 data] | NA | NA | 1.17 (1.11, 1.23) | 1.15 (1.04, 1.27) |  |  |
| **Gore et al., 2012** | In-hospital [2002 data] | NA | NA | 1.12 (1.04, 1.21) | 1.12 (1.04, 1.21) |  |  |
| **Gore et al., 2012** | In-hospital [2006 data] | NA | NA | 1.13 (1.00, 1.28) | 1.04 (0.91, 1.19) |  |  |
| **Greenland et al., 1991** | In-hospital | NA | NA | 1.25 (0.97, 1.59) | 1.91 (1.43, 2.55) | - Demographics (Age)  - CVD risk factors (hypertension)  - AMI-related characteristics (Previous MI, AV block, anterior MI)  - Other clinical characteristics (Heart failure) | Self-reported |
| **Koek et al., 2007** | 28 days post discharge | 1.52 (1.32, 1.76) | 1.18 (1.04, 1.33) | 1.16 (0.99, 1.36) | 1.12 (0.97, 1.30) | - Demographics (Age, ethnic, gender)  - CVD risk factors (Previous CVD) | Patients with history of DM registered during previous hospital admissions in  1991–1995 or during the index  admission in 1995 |
| **Lopez-de-Andres et al., 2021** | In-hospital: USTEMI | 5.40 (4.89, 5.95) | 1.83 (1.63, 2.05) | NA | NA | None | Diabetes defined by the American Diabetes Association criteria |
| **Lopez-de-Andres et al., 2021** | In-hospital: STEMI | 3.15 (2.91, 3.40) | 2.69 (2.47, 2.92) | NA | NA | None |  |
| **Meisinger et al., 2010** | 28 days post-discharge | 1.59 (1.12, 2.26) | 1.79 (1.04, 3.08) | NA | NA | None | Patient interview during hospital stay |
| **Miettinen et al., 1998** | 28 days post-discharge | NA | NA | 1.56 (1.14, 2.15) | 2.57 (1.69, 3.91) | - Demographics (Age, study area) | Patient interview, medical record review, receiving insulin or oral hypoglycemic treatment |
| **Zuanetti et* al., 1993** | In hospital: Insulin treatment | 2.83 (1.35, 5.95) | 1.69 (1.24, 2.30) | NA | NA | None | Patient history collected during in-hospital stay |
| **Zuanetti et* al., 1993** | In hospital: Oral diabetic medication | 3.43 (2.33, 5.05) | 1.14 (0.89, 1.46) | NA | NA | None |  |
| **Sex-specific mid-term mortality** | | | | | | | |
| **Abbud et al., 1995** | 3 years | 1.30 (1.25, 1.35) | 1.12 (1.08, 1.16) | NA | NA | None | Medical record |
| **Ahmadi et al., 2015** | 1 year | NA | NA | 1.39 (1.23, 1.57) | 0.98 (0.83, 1.15) | Clinical & demographic risk factors | Fasting plasma glucose (FPG) ≥7 mmol/L, self-report of diagnosis verified by a physician and on medication |
| **Behar et al., 1997** | 1 year: Insulin treatment | 2.73 (1.14, 6.56) | 6.81 (3.00, 15.42) | NA | NA | None | History of diabetes, physician diagnosis |
| **Blondal et al., 2012** | 5 years | NA | NA | 1.20 (0.81, 1.78) | 1.83 (1.17, 2.86) | - Demographics (Age)  - CVD risk factors (Current smoking status, previous AMI)  - AMI-related characteristics (Previous PCI, previous CABG. STEMI, Killip III-IV on admission; LVEF <40%, 3-4 vessel disease)  - Other clinical characteristics (arterial hypertension, dyslipidemia, chronic heart failure, previous stroke, peripheral vascular disease, delay to FMC) | Medical history documented by physician, diagnosed during current episode |
| **Brophy et al., 2010** | 90 days post-discharge | 1.57 (1.52, 1.62) | 1.08 (1.05, 1.11) | NA | NA | None | ICD codes E10-E14 (Insulin dependent, non-insulin dependent, malnutrition-related diabetes mellitus, other specified diabetes, and other non-specified diabetes) |
| **Brophy et al., 2010** | 6 months | 1.80 (1.66, 1.96) | 1.50 (1.35, 1.66) | NA | NA | None |  |
| **Brophy et al., 2010** | 1 year | 1.08 (0.97, 1.21) | 1.50 (1.35, 1.66) | NA | NA | None |  |
| **Greenland et al., 1991** | 1 year | NA | NA | 0.95 (0.68, 1.32) | 1.67 (1.10, 2.54) | - Demographics (Age)  - CVD risk factors (Diabetes, cigarette smoking, previous MI)  - Other clinical characteristics (Angina before MI, hypertension before MI) | Self-reported |
| **Koek et al., 2007** | 1 year | 1.74 (1.57, 1.93) | 1.31 (1.19, 1.43) | 1.33 (1.12, 1.58) | 1.23 (1.09, 1.39) | - Demographics (Age, native ethnic origin)  - Other clinical characteristics (Prior admission for cardiovascular disease, type of hospital, length of stay) | Patients with history of DM registered during previous hospital admissions |
| **Koek et al., 2007** | 5 years | 1.75 (1.64, 1.86) | 1.39 (1.31, 1.47) | 1.49 (1.36, 1.63) | 1.39 (1.12, 1.20) |  |  |
| **Meisinger et al., 2010** | 4 years | 1.67 (1.37, 2.04) | 2.39 (1.75, 3.26) | NA | NA | None | Patient interview during hospital stay |
| **Miettinen et al., 1998** | 1 year | NA | NA | 1.38 (1.18, 1.61) | 1.85 (1.39, 2.46) | - Demographics (Age, study area) | Patient interview, medical record review, receiving insulin or oral hypoglycemic treatment |
| **Mukamal et al., 2001** | 3.7 years | NA | NA | 1.29 (0.99, 1.68) | 2.66 (1.78, 4.00) | - Demographics (Age, sex, household income (in quartiles), education (in three categories),  - CVD risk factors (Current smoking, previous smoking)  - AMI-related characteristics (  previous MI, Angina, use of thrombolytic therapy, usual frequency of exertion),  - Other clinical characteristics (hypertension, medication use before hospitalization (aspirin, b-adrenergic antagonists, calcium-channel blockers, Angiotensin-converting-enzyme inhibitors, BMI, alcohol consumption, complications of congestive heart failure or ventricular tachycardia during hospitalization) | History of diabetes obtained during chart review, current use of any hypoglycemic medication |
| **Muller et al., 2004** | 5 years | NA | NA | 2.09 (1.27, 3.44) | 0.95 (0.39, 2.32) | Baseline characteristics, ECG findings, markers of inflammation and myocardial necrosis, angiographic extent of coronary artery disease | Patients with a known history of DM treated currently with diet intervention, oral glucose-lowering agents, or insulin |
| **Norhammar et al., 2003** | 1 year | 3.29 (2.56, 4.23) | 3.54 (2.58, 4.86) | NA | NA | None | Patient information at hospital admission, prescription of insulin or oral anti-diabetic drugs at admission and/or at discharge |
| **Schramm et al., 2008** | 5 years | NA | NA | 1.39 (1.33, 1.45) | 1.16 (1.12, 1.20) | - Demographics (Age, gross income)  - Other clinical characteristics (Comorbidities) | At least 1 prescription of glucose-lowering medication (oral or insulin) in the 6-month period before January 1, 1997 |
| **Zuanetti et al., 1993*** | 6 months: Insulin treatment | 1.40 (0.52, 3.72) | 3.20 (2.03, 5.04) | NA | NA | None | Medical history collected during in-hospital stay |
| **Zuanetti et al., 1993*** | 6 months: Oral diabetic medication | 2.60 (1.68, 4.02) | 1.24 (0.78, 1.96) | NA | NA | None |  |
| **Sex-specific long-term mortality** | | | | | | | |
| **Abbott et al., 1988** | 34 years | NA | NA | 1.11 (0.60, 2.03) | 1.83 (0.91, 3.66) | - Demographics (Age)  - CVD risk factors (Total cholesterol level, cigarette usage)  - Other clinical characteristics (Hypertension status, body mass index, cardiac failure, left ventricular hypertrophy, oral or insulin treatment) | History of  treatment with insulin or oral hypoglycemic agents, abnormal results of glucose tolerance tests, casual blood  glucose concentrations of 8.3 mmol/L or more on at least two Framingham examinations |
| **Behar et al., 1997** | 10 years: Insulin treatment | NA | NA | 1.78 (1.29, 2.47) | 2.58 (1.89, 3.54) | - Demographics (Age)  - AMI related characteristics (Previous myocardial infarction, previous angina, anterior myocardial infarction site. serum lactate dehydrogenase levels)  - Other clinical characteristics (Hypertension, congestive heart failure, ventricular tachycardia, and advanced atrioventricular block occurring during hospitalization) | History of diabetes, physician diagnosis |
| **Behar et al., 1997** | 10 years: Oral diabetic medication | NA | NA | 1.33 (1.12, 1.58) | 1.41 (1.10, 1.80) |  |  |
| **Hu et al., 2005** | 7.7 years | NA | NA | 2.07 (1.56, 2.75) | 1.44 (0.94, 2.20) | - Demographics (Age, study year)  - CVD risk factors (Systolic blood pressure, total cholesterol, current smoking status)  - Other clinical characteristics (BMI) | Self-reported |

Abbreviations:

NA: not available

AV block: atrioventricular block

MI: myocardial infarction

STEMI: ST-elevation myocardial infarction

CABG: coronary artery bypass graft surgery

PCI: Percutaneous coronary intervention

LVEF: ejection fraction

BMI: body mass index

*: indicating randomized control study
